# Identification and validation of novel biomarker TRIM8 related to cervical cancer

**DOI:** 10.3389/fonc.2022.1002040

**Published:** 2022-10-24

**Authors:** Li Zhang, Youli Dan, Chaoyang Ou, Hongyan Qian, Yi Yin, Min Tang, Qian He, Chen Peng, Aiqin He

**Affiliations:** ^1^ Department of Cancer Research Center, Nantong Tumor Hospital, The Affiliated Tumor Hospital of Nantong University, Nantong, China; ^2^ Department of Gynecology Oncology, Nantong Tumor Hospital, The Affiliated Tumor Hospital of Nantong University, Nantong, China; ^3^ Department of Clinical Laboratory Diagnostics, Nantong Tumor Hospital, The Affiliated Tumor Hospital of Nantong University, Nantong, China; ^4^ Department of Gynecology and Obstetrics, The Affiliated Hospital of Nantong University, Nantong, China

**Keywords:** cervical cancer, GEO, bioinformatics, TRIM8, biomarker

## Abstract

**Background:**

Cervical cancer, as a common gynecological disease, endangers female health. Give the lack of effective biomarkers for the diagnosis and treatment of cervical cancer, this paper aims to analyze the Gene Expression Omnibus (GEO) data sets using comprehensive bioinformatics tools, and to identify biomarkers associated with the cancer in patient samples.

**Methods:**

The bioinformatics methods were used to extract genes related to cervical cancer from GSE39001, while the GEO2R online tool to elaborate on differentially expressed genes (DEGs) in normal and cancer samples, and to clarify related genes and functions. The results were verified by IHC, WB, CCK-8, clone formation and flow cytometry experiments.

**Results:**

A total of 2,859 DEGs were identified in the GEO microarray dataset. We extracted genes associated with both ubiquitination and autophagy from the key modules of weighted gene co-expression network analysis (WGCNA), and the analysis showed that TRIM8 was of great significance for the diagnosis and prognosis of cervical cancer. Besides, experimental validation showed the high TRIM8 expression in cervical cancer, as well as its involvement in the proliferation of cervical cancer cells.

**Conclusion:**

We identified a biomarker (TRIM8) that may be related to cervical cancer through a series of analyses on the GEO dataset. Experimental verification confirmed the inhibition of cervical cancer cells proliferation by lowering TRIM8 expression. Therefore, TRIM8 can be adopted as a new biomarker of cervical cancer to develop new therapeutic targets.

## Introduction

As one of the most common gynecological malignancies, cervical cancer is the fourth leading cause of new cancer and death among women in the world. In 2021, there were 600,000 new cases and 340,000 deaths of cervical cancer worldwide (1, 2). Its evolution is a multi-step biological process triggered by a series of genetic and transcriptional changes. Human papillomavirus (HPV) infection is considered to be the main cause of cervical cancer (3). Despite the leap in screening, the 5-year overall survival rate of cervical cancer remains about 60% (4). Besides, the application of HPV preventive vaccine is seriously limited in many regions, especially in low-and middle-income countries, due to the high cost and low coverage (5). Most patients were diagnosed as cervical cancer at an advanced stage caused by the lack of early diagnosis, missed the optimal surgery opportunity, and often became resistant to radio/chemotherapy, resulting in high mortality (6–8). Therefore, identifying specific biomarkers remains urgent for better accuracy of diagnosis.

High throughput technology has become one of the most important methods in genomics research. Thanks to their extensive application in clinical gene detection, especially in malignant tumors, microarray and sequencing technologies are vital for the diagnosis, treatment, recurrence monitoring, and drug resistance mechanism of cancer patients (9, 10). The derivation of multi omics public databases, such as GEO and The Cancer Genome Atlas (TCGA), has made it a trend for researchers to analyze microarray data and compare cancer atlas with normal atlas (11, 12). In the research of cervical cancer, bioinformatics analysis is used to identify biomarkers and signal pathways related to the disease progression. Through a comprehensive bioinformatics analysis from GEO datasets, Zhu et al. identified TCAM1P as a cancer/testis pseudogene regulated by HPV E6/E7 and EIF4A3, as well as its role as a promoter to promote the proliferation of cervical cancer cells (13). Li et al. analyzed bioinformatics, and revealed hsa-miR-504’s close relation with the occurrence and development of cervical cancer, as well as its role as a potential molecular marker for the prognosis of the cancer (14). In addition, some researchers calculated the immune and stromal scores of cervical cancer patients in TCGA database, elaborated DNA promoter methylation and gene expression, and identified the tumor microenvironment and DNA methylation related prognostic characteristics, which can accurately predict the prognosis, and provide ideas for hierarchical management and targeted treatment (15). The public microarray database of cervical cancer, however, requires more exploration, which promotes the identification of potential biomarkers related to the disease progression.

This study extracted DEGs from the microarray dataset GSE39001 to construct gene module of DEGs, and to screen the key genes within. The hub gene related to ubiquitination and autophagy was also obtained. In this way, the E3 Ubiquitin-Protein Ligase TRIM8 was screened. Then, we used the online database to further explore TRIM8, evaluated the potential therapeutic drugs, and verified the biological role of TRIM8 in cervical cancer cells through cell function experiments. Finally, the possible upstream and downstream regulation mechanisms of TRIM8 were evaluated based on large public databases. This result may provide new insights for the diagnosis and prognosis of cervical cancer.

## Materials and methods

### Microarray data collection

In order to find the gene chip data related to cervical cancer, “cervical cancer” was input as the keyword to obtain related data set through the GEO module in the National Center for Biotechnology Information (NCBI), and the GSE39001 microarray data set was downloaded. The platform for GSE39001 is GPL201, [HG-Focus] Affymetrix Human HG-Focus Target Array, which includes 12 normal cervical samples and 43 cervical cancer tissues.

### Identification of DEGs and functional enrichment analysis

We utilized GEO2R (https://www.ncbi.nlm.nih.gov/geo/geo2r/?acc=GSE39001) online website to analyze all genes of normal and cancer samples, and downloaded corresponding files to screen DEGs. The DEGs were selected based on the adjustment p value <0.05 and | log fold change (FC) | >0.5.

Through the cluster profiler package in R, the Gene Ontology (GO) and Kyoto Encyclopedia of Genes and Genomes (KEGG) pathways of DEGs were enriched and analyzed to determine their potential functions and pathways. GO analysis, which mainly includes three aspects of biological processes, cellular components, and molecular functions, helped to understand the biological functions, pathways, or positioning of DEGs. KEGG pathway analysis clarified the signal pathways that DEGs may focus on. P<0.05 was considered to be statistically significant.

### Weighted gene co-expression network analysis

We use the R package of “WGCNA” to construct the co-expression network of DEGs, and create an adjacency matrix to describe the correlation strength between nodes. When the scale-free R^2^ is 0.87, the soft-threshold β=10 is selected, and the adjacency matrix is transformed into a topological overlap matrix (TOM). Next, hierarchical clustering is performed to identify modules, each of which contains at least 30 genes with a sensitivity of 3 and a module merging threshold of 0.25. Finally, the characteristic genes are calculated, the modules clustered hierarchically, and similar modules merged.

The module feature association is evaluated by the correlation between module co-expressed genes and clinical features, which makes it easy to identify the expression sets (modules) highly correlated with clinical features. For each gene expression profile, the gene significance (GS) is calculated as the absolute value of the correlation between the gene and each clinical feature. Module membership (MM), defined as the correlation between genes and characteristic genes of each module, describes the reliability of genes belonging to modules. In this study, the threshold value of GS and MM was set to be 0.1 and 0.5, respectively.

### Screening of genes and construction of protein-protein interaction network

Keywords of “ubiquitination” and “autophagy” were input in the molecular signatures database module in Gene Set Enrichment Analysis (GSEA) (http://www.gsea-msigdb.org/gsea/index.jsp) to obtain the needed gene sets, thus extracting the needed genes. Two gene sets named M3309 and M15852 were downloaded. Then, the genes and module genes were used to draw the overlapping gene Venn diagrams using the online website dedicated to making Venn diagrams (http://bioinformatics.psb.ugent.be/webtools/Venn/).

STRING (https://cn.string-db.org/) online database is employed to clarify the interaction between different proteins, thus constructing PPI network. The database is a computerized and powerful global resource tool for validating the interacting proteins found in predictions and experiments. This paper also uses Cytoscape software (version 3.8.2) for visual analysis, Spearman’s correlation statistical method for the correlation between genes, and R package corrplot (version 0.84) for the multi gene correlation map. Based on these genes, R package GOplot and ggplot2 are adopted for function enrichment and visual analysis.

### Cancer stem cells score and mutation analysis

The CSCs score was obtained according to the one-class logistic regression (OCLR) algorithm constructed by Malta et al. (16). The RNAseq data and corresponding clinical information of 306 cervical cancer patients were obtained from TCGA dataset. The OCLR algorithm was used to calculate the mRNA stemness index (Si). Based on the characteristics of mRNA expression, the gene expression profile of 11,774 genes was included. Besides, this paper employed the Spearman correlation, and mapped the Si to the (0, 1) range by subtracting the minimum value and dividing it by the maximum value. All analysis methods and R packages were implemented using v4.0.3 R software (R foundation for statistical computing, 2020).

The somatic mutation data of cervical cancer patients were obtained from TCGA dataset (https://portal.gdc.com). The maftools package in R software was taken to download and extract mutation data, and the somatic mutation data of patients in high and low gene expression groups were then visually analyzed.

### Correlated genes of TRIM8 in cervical cancer

The LinkedOmics database contains multiomics data and clinical data of 32 cancer types, as well as data of 11,158 patients in the TCGA project, integrating proteomic data from mass spectrometry (MS) of selected TCGA tumor samples. 304 TCGA-CESC samples were picked for analysis. Based on DEGs mined from LinkedOmics database, R package clusterprofiler and ggplot2 were used for function enrichment and visual analysis.

### Drug sensitivity analysis

As a repository that integrates numerous chemical substances, genes, functional phenotypes, and interactions between diseases, the Gene Set Cancer Analysis (GSCA) (http://bioinfo.life.hust.edu.cn/GSCA/#/) facilitates the study of disease-related environmental exposure factors and potential drug action mechanisms, and provides information on tumor drug response data and genome sensitive markers. The structure of small molecule compounds is obtained through the National Library of Medicine (NLM) online website (https://pubchem.ncbi.nlm.nih.gov/).

### Single-sample gene set enrichment analysis

This paper collected the genes in the corresponding pathways, adopted R package GSVA for analysis, selected the parameter method=‘ssgsea’, and analyzed the correlation between genes and pathway scores through Spearman correlation. P<0.05 was considered statistically significant.

### Query for E3-TRIM8 interactions in UbiBrowser

The UbiBrowser (http://ubibrowser.bio-it.cn/ubibrowser_v3/) is an integrated bioinformatics platform, which can systematically analyze the protein network, structure and sequence involved in the interaction between ubiquitin ligase and substrate, so as to investigate ubiquitin ligase substrate interaction networks. This paper also took the UbiBrowser to predict the potential substrate of TRIM8 interaction, and Cytoscape to conduct visual analysis.

### Gene correlation analysis and miRNA prediction

We used the “correlation analysis” module in GEPIA2 online website (http://gepia2.cancer-pku.cn/#correlation) to perform Spearman correlation analysis on the substrates interacting with TRIM8. The log_2_ TPM was adopted for the dot chart, and P value and correlation coefficient (R) were exhibited.

The miRNA prediction is made based on miRDB online database (http://mirdb.org/mirdb/index.html), which is developed for miRNA target prediction and functional annotation by analyzing the interaction of miRNA targets from high-throughput sequencing experiments. Besides, visual analysis was carried out through Cytoscape.

### Patients

The paraffin specimens of tissue microarray used are provided by Nantong Tumor Hospital. Patients with stage IA2-IIA2 cervical cancer who first visited Nantong Tumor Hospital from June 2008 to September 2014 were randomly selected. The clinicopathological data of patients (such as age, pathological type, FIGO stage, degree of differentiation, tumor size, depth of interstitial invasion, vascular invasion, lymph node metastasis, and postoperative adjuvant therapy) were collected and followed up. Incomplete clinicopathological data or lost patients with cervical cancer were excluded. Finally, 176 patients with cervical cancer met the inclusion criteria. The inclusion criteria are as follows: (1) All patients underwent radical hysterectomy without neoadjuvant therapy before operation. Postoperative adjuvant therapy is considered according to pathological risk factors. (2) There was no complication or secondary malignant tumor. (3) The patients had complete clinicopathological data and follow-up results, and were followed up to August 2020. (4) All cases were confirmed as cervical cancer by histology. This study is approved by the ethics committee of Nantong Tumor Hospital (No.2022-A 08), and all procedures are carried out following the Guidelines of the World Medical Association Declaration of Helsinki.

### Immunohistochemistry

The cancerous tissues and corresponding paracancerous tissues from the 176 subjects with cervical cancer were pooled, and tissue microarrays were made by pathologists. The cut tissue chip was placed on the paraffin slicer to bake, immersed in xylene for dewaxing, and hydrated in graded absolute ethanol. It was sliced and added with citrate buffer solution under pressure cooker, and boiled for 3 min to extract antigen. After the slices were washed with PBS solution, 3% hydrogen peroxide solution was added to remove endogenous peroxidase. After incubation at room temperature, they were washed with PBS. Tissue chip sections were placed on a wet box and incubated with TRIM8 antibody (ab155674, Abcam, UK) at 4 °C overnight. The next day, the slices were washed and incubated with secondary antibody (PV-6001, Zhongshan Biotech, Beijing, China) at room temperature. The washed sections were then incubated with DAB (Dako, DK) in the dark, re-stained with hematoxylin, dehydrated with absolute ethanol, and finally fixed with neutral resin. TRIM8 staining scores in tissue microarray were calculated by two pathologists independently and double-blind, and the dyeing intensity of TRIM8 was analyzed by semi quantitative method. Immunostaining intensity: 0 point for no response; 1 point for weak response; 2 points for moderate response; and 3 points for strong response. Proportion: 0-25 points for 0-25%, 26-50 points for 26-50%, 51-75 points for 51-75% and 76-100 points for 76-100%. The score is calculated as follows: final score = (0 × Percent no response) + (1 × Weak response percentage) + (2 × Moderate percentage of response) + (3 × Reaction intensity percentage). Then, the samples were divided into two groups according to the final score: low expression (tissue score from 0 to 180), and high expression (tissue score from 181 to 300).

### Cell culture and transfection

The two human cervical cancer cell lines HeLa and SiHa involved in this study were purchased from the cell bank of Chinese Academy of Sciences. The cells were cultured in DMEM (HyClone, USA) medium containing 10% fetal bovine serum (FBS, Gibco, USA) at 37 °C in a constant temperature environment containing 5% CO_2_.

In this study, small interfering RNA (siRNA) was used as the vector for transfection, and the instructions of siRNA kit were referred for transfection. When the fusion degree of HeLa and SiHa cells reached 70%-80% and they were in logarithmic growth phase, Lipofectamine 2000 (Thermo Fisher Scientific) liposomes was used to transfect with siRNA against TRIM8 (siTRIM8) or scrambled siRNA (siNC). In order to ensure the jamming efficiency of the target, three siTRIM8 sequences were designed (human siTRIM8-1: 5’-ACACCAAGUCUGUGAAAAUTT-3’; human siTRIM8-2: 5’-CGUGGAGAUCCGAAGGAAUTT-3’; and human siTRIM8-3: 5’-AGACGGAGGAUGUCAGCUUTT-3’).

### Survival analysis

In order to clarify the relation between TRIM8 expression with the prognosis of cervical cancer, cervical cancer specimens were categorized into high expression and low expression groups according to the pathological score of TRIM8, and the K-M curve was drawn. Both overall survival and cervical cancer specific survival rate were used as the end points. The SPSS (version 25.0) software was taken to evaluate the prognostic value of TRIM8 in cervical cancer. P<0.05 was considered statistically significant.

### Immunofluorescence

In a 24-well plate, the small round glass sheets carrying cells were washed with PBS, the cells were fixed with 4% paraformaldehyde, and then the residual paraformaldehyde solution was washed with PBS. At the same time, 0.2% Triton X-100 was prepared to permeate the cells at room temperature. The glass sheets were soaked with PBS, and 1% BSA sealing solution was added. After discarding the liquid, a sufficient amount of diluted TRIM8 specific antibody (ab155674, Abcam, UK) was added to each glass sheet and placed in a wet box for incubation at 4° C overnight. The next day, the glass was washed with PBS, and the diluted fluorescent secondary antibody (ab150077, Abcam, UK) was added dropwise, incubated at room temperature in dark for 1 h, and then the glass was soaked with PBS. The DAPI (Beyotime Biotechnology, Shanghai, China) was added dropwise in the dark to re-stain the nuclei for 5 min, and the extra DAPI was washed away with PBS. The glass slide was taken out, buckled upside down on the slide, and added with the sealing liquid, and the image was collected under the fluorescence microscope.

### Western blot

The radio immunoprecipitation assay (RIPA) (Beyotime Biotechnology, Shanghai, China) lysis buffer supplemented with PMSF and protease inhibitor was used to extract total cellular proteins and quantify the protein concentration. The proteins were separated by sodium dodecyl sulfate–polyacrylamide gel electrophoresis (8% SDS-PAGE) (Beyotime Biotechnology, Shanghai, China), transferred to polyvinylidene fluoride (PVDF) (Millipore, Temecula, CA, USA) membrane, and blocked with 5% not-fat milk at room temperature. The membrane was sealed with diluted TRIM8 specific antibody (ab155674, Abcam, UK) overnight at 4 °C, cleaned and incubated with secondary antibody (A0208, Beyotime Biotechnology, Shanghai, China) at room temperature. After cleaning the membrane, the enhanced chemiluminescence (ECL) (Tanon, Shanghai, China) was used to detect protein bands.

### Cell proliferation assay

Cell proliferation was assessed by CCK-8 (Vazyme, Nanjing, China) analysis. Transfected HeLa and SiHa cells were inoculated into 96-well plates in advance, and 10 μL CCK-8 solution was added to each well, and incubate at 37°C for 2 h. The cell proliferation was assessed by detecting OD450 with an automatic spectrophotometer.

The transfected HeLa and SiHa cells were inoculated into 6-well plates with 2000 cells per well to form cell clones. After 14 days of culture, they were fixed with 4% paraformaldehyde, and stained with crystal violet. The number of cell colonies in each plate was later calculated.

The transfected HeLa and SiHa cells were collected into a centrifuge tube, resuspended with binding buffer washing solution for 3 times, and stained with PI at room temperature and in dark for 15 minutes. Besides, the samples were detected by flow cytometry.

### Statistical analysis

SPSS 25.0 software was used for statistical analysis, while Graphpad Prism 8 software for drawing the survival curve and statistical chart of the difference between the two groups. The difference was tested by independent sample t-test or paired sample test, and the correlation between TRIM8 expression and clinicopathological parameters was tested by χ2 test. In addition, Cox proportional hazards regression model was taken for univariate and multivariate analysis, while Kaplan Meier method and Log-rank test for overall survival analysis. P<0.05 was statistically significant.

## Results

### Identification of DEGs and functional enrichment analysis

**Figure 1:**
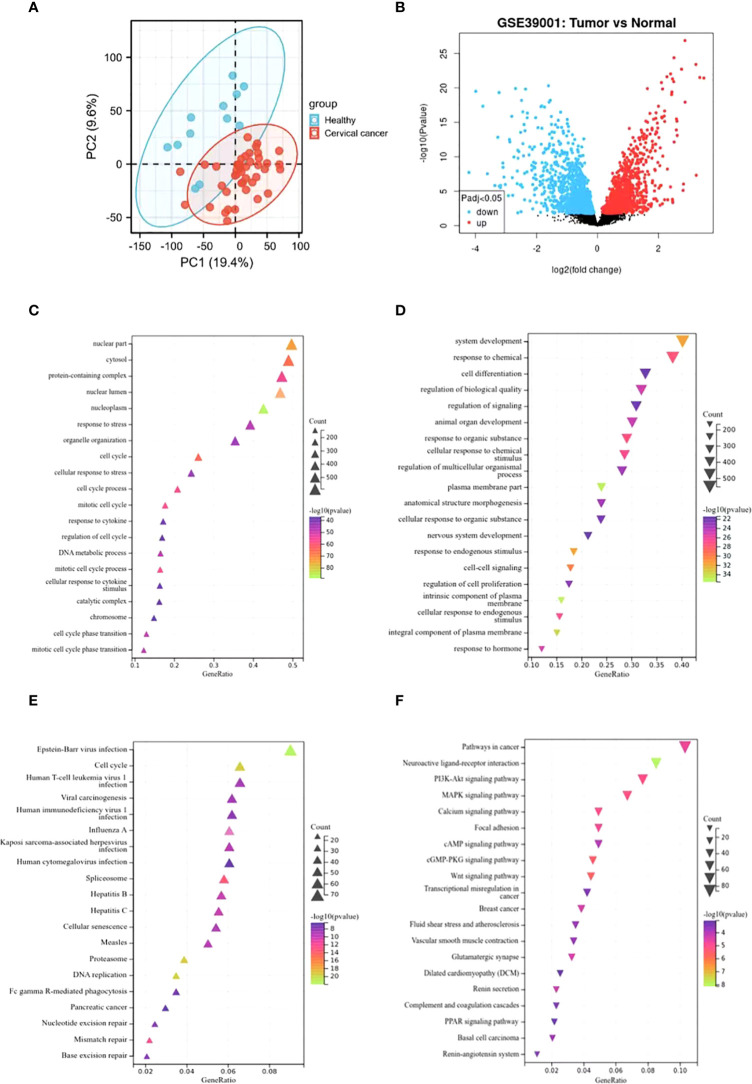
Identification of DEGs in GSE39001 and functional enrichment analysis. **(A)** Principal component analysis for GSE39001. **(B)** Volcano plot of the 2,859 DEGs. The red dots represent the significantly up-regulated genes, and the blue dots the significantly down-regulated genes. **(C, D)** GO analysis of high and low expression DEGs, respectively. **(E, F)** KEGG analysis of high and low expression DEGs, respectively.

The principal component analysis (PCA) was performed to evaluate the repeatability of the data within the group, confirming the favorable repeatability of the data in GSE39001 (**Figure 1A**). GEO2R online website was also employed to analyze the gene expression data between 12 normal cervical tissues and 43 primary cervical tumor tissues in GSE39001. A total of 2,859 DEGs were identified, including 1,538 up-regulated and 1,321 down-regulated DEGs, which were displayed by volcanic map (**Supplementary Table 1**). Specifically, red and blue represented up-regulated and down-regulated genes, respectively (**Figure 1B**), both of which were analyzed by GO and KEGG enrichment. GO analysis revealed that the up-regulated DEGs was mainly related to the biological process of the nucleus (**Figure 1C**), while the down-regulated DEGs was mainly enriched in the biological process related to the tumor (**Figure 1D**). KEGG analysis showed that the up-regulated DEGs was mainly related to cell cycle and virus infection (**Figure 1E**), while the down-regulated DEGs was mainly concentrated in the signal pathway related to tumor progression (**Figure 1F**).

### WGCNA Co-expression network

**Figure 2:**
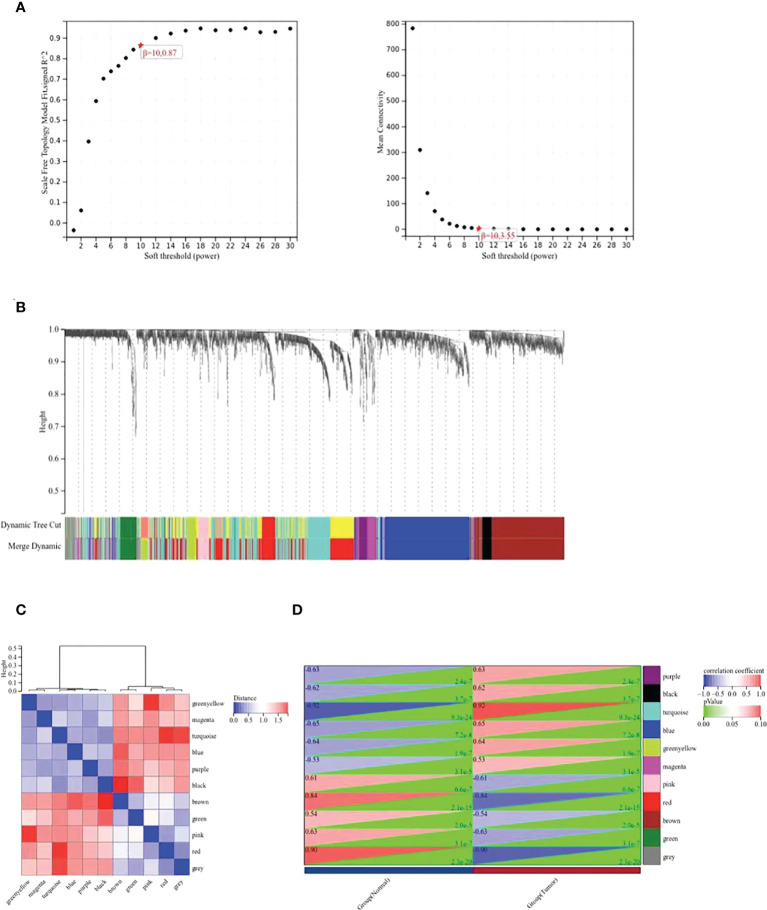
WGCNA co-expression network analysis of GSE39001. **(A)** Analysis of the scale-free index and the mean connectivity for various soft-threshold powers (β). **(B)** Dendrogram of all differentially expressed genes clustered based on the measurement of dissimilarity. The color band shows the results obtained from the automatic single-block analysis. **(C)** Modular eigenvector clustering. **(D)** Correlation between modules and clinical trait according to Pearson correlation.

The WGCNA co-expression network was constructed based on the DEGs in GSE39001. As shown in **Figure 2A**, the soft threshold of β=10 (R^2^ = 0.87) to build a scale-free network. All DEGs were divided into co-expression modules of ten different genes, and displayed in various colors (**Figure 2B**). **Figure 2C** plots the specific module eigenvector clustering. According to the correlation between module characteristic genes, and clinical features, the top two modules most relevant to cervical cancer were collected for further analysis (**Figure 2D**).

### PPI network and analysis of the differentially expressed ubiquitination-related genes

**Figure 3:**
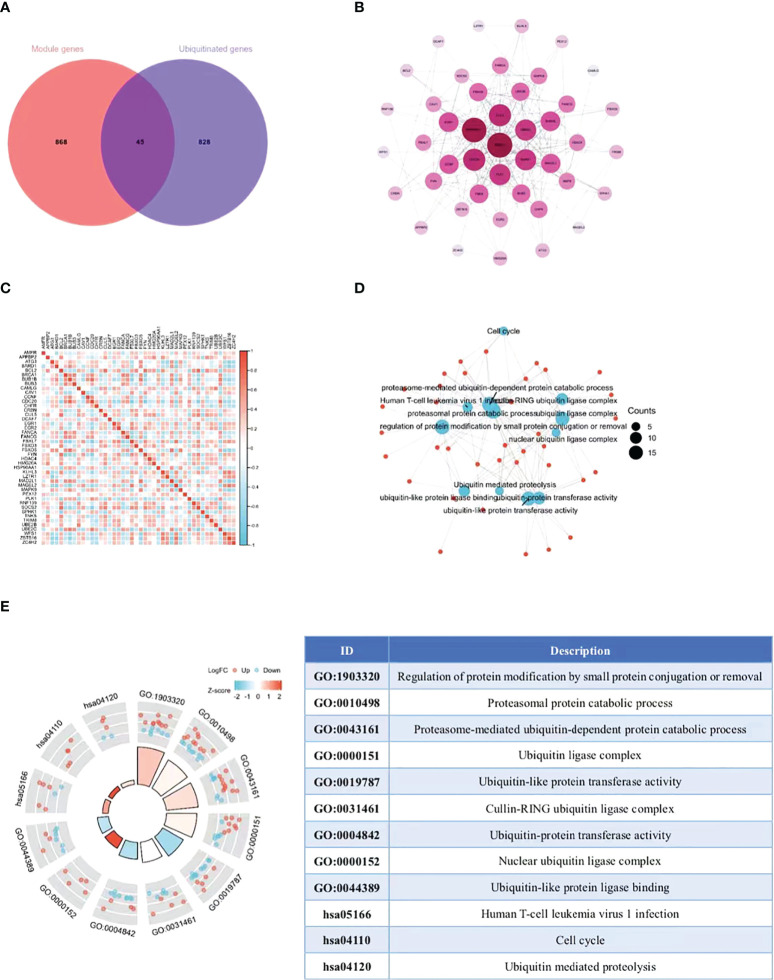
Extraction and analysis of ubiquitination-related genes. **(A)** The Venn diagram of module genes and ubiquitination-related genes. **(B)** The PPI network of 44 eligible genes. The darker the color, the higher the connectivity of genes. **(C)** Spearman correlation analysis of the 40 differentially expressed ubiquitination-related genes. **(D, E)** GO and KEGG analysis of the 40 differentially expressed ubiquitination-related genes.

The ubiquitin-proteasome pathway affects the occurrence and development of malignant tumors by participating in the regulation of various cellular biological processes. The research on molecular mechanism of this pathway exhibits prospects and gains popularity inside and outside China in recent years (17). At present, the research on the role of ubiquitin-proteasome pathway in cervical cancer is still in its infancy. On this basis, we collected the overlap points of 913 module key genes in the top two most relevant modules of WGCNA and ubiquitination-related genes (**Supplementary Table 2**). As illustrated in the Venn diagram, a total of 45 eligible genes were identified (**Figure 3A**). In order to determine the interaction between differentially expressed ubiquitination-related genes, Cytoscape software was utilized for PPI network analysis, which discovered the interation among 44 ubiquitination-related genes (**Figure 3B**). Subsequently, the expression correlation of such genes was analyzed to prove the relationship between the 44 differentially expressed ubiquitination-related genes in the GSE39001 dataset (**Figure 3C**). To determine their potential biological functions, R software was adopted for GO and KEGG enrichment analysis, which confirmed the involvement of the most important GO enrichment items in regulating protein modification by methods including small protein conjugation or removal, proteasomal protein catabolic process, proteasome mediated ubiquitin dependent protein catabolic process (biological processes), ubiquitin ligase complex, cullin-RING ubiquitin ligase complex, nuclear ubiquitin ligase complex (cellular component), ubiquitin-like protein transferase activity, ubiquitin-protein transferase activity, and ubiquitin-like protein ligase binding (molecular function). KEGG enrichment analysis validated the involvement of such genes were in human T-cell leukemia virus 1 infection, cell cycle, and ubiquitin mediated proteolysis (**Figures 3D, E**).

### Identification and analysis of key gene TRIM8

**Figure 4:**
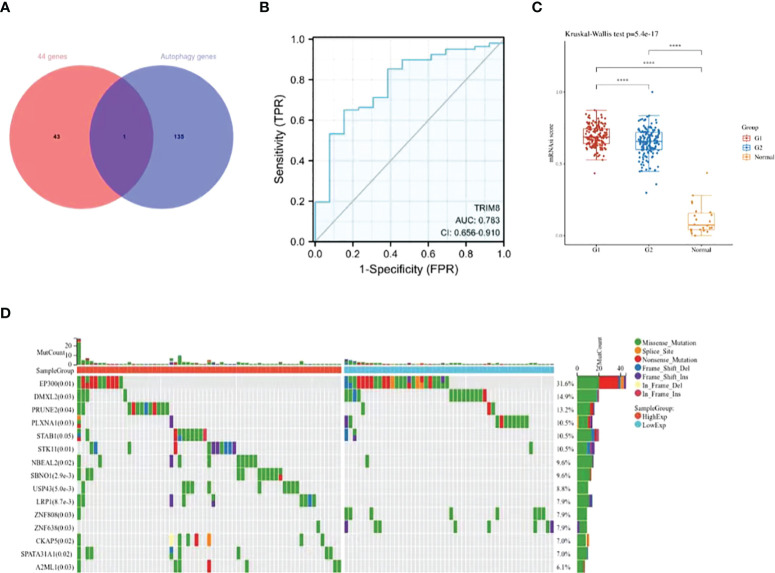
Identification and analysis of TRIM8. **(A)** The Venn diagram of 44 ubiquitination-related genes and autophagy -related genes. **(B)** The ROC curve of TCGA-CESC according to the expression of TRIM8. **(C)** Distribution of CSCs scores in different groups. G1 represents the low expression group of TRIM8, and G2 the high expression group of TRIM8. ****p < 0.001. **(D)** The top 15 mutation genes in high and low TRIM8 expression group, respectively.

Despite the proved role of autophagy in the occurrence and development of cervical cancer (18), more efforts are required to fill the research blank on the specific mechanism. In this way, more autophagy-related genes and corresponding signal pathways will be available for the diagnosis and treatment of cervical cancer. Therefore, we collected 44 overlapping points of differentially expressed ubiquitination-related genes and autophagy-related genes (**Supplementary Table 2**), and obtained a key gene TRIM8 (**Figure 4A**). ROC curve was used to verify the prognostic ability of TRIM8 in TCGA-CESC cohort. **Figure 4B** reveals the AUC of TRIM8 to be 0.783 (p<0.001). Combined with high sensitivity and specificity, TRIM8 can serve as a candidate prognostic biomarker for cervical cancer. In addition, the CSCs score proved the better cancer stem cell characteristics of the TRIM8 high expression group compared with the normal control group (**Figure 4C**). According to the gene mutation landscape map, significant mutations were observed among EP300, PRUNE2, STAB1, STK11, and SBNO1 genes in the high TRIM8 expression group, while among EP300, DMXL2, PLXNA1, ZNF808 and ZNF638 genes (**Figure 4D**) in the low TRIM8 expression group.

### Genes correlated with TRIM8 in cervical cancer

**Figure 5:**
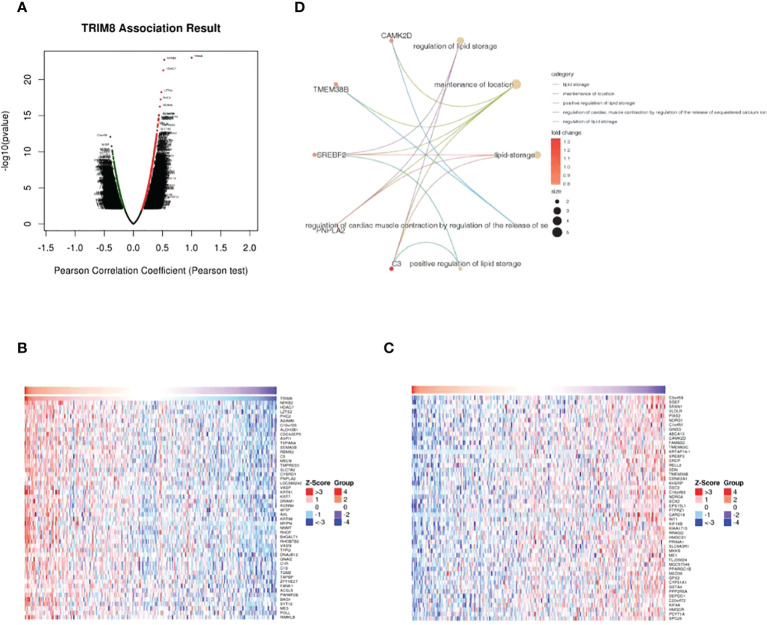
Genes associated with TRIM8 expression in cervical cancer. **(A)** Correlations between TRIM8 and differently expressed genes from LinkedOmics database. **(B, C)** Heat maps show the genes that are positively or negatively correlated with TRIM8 (Top 50 genes). **(D)** GO enrichment of top 20 positive and negative candidate genes.

LinkedOmics database was adopted to evaluate the TRIM8 related genes in cervical cancer. The volcanic map in **Figure 5A** reveals that the positively related genes converge on the right side of 0 (positive values), while the negatively related genes on the left side (negative values). According to the Spearman test, the top 50 positively and negatively related genes were identified and analyzed as shown in the heat maps (**Figures 5B, C**). Then, top 20 genes with positive and negative correlation were selected, respectively, and conducted with GO enrichment analysis, which certified the role of the most important GO enrichment items in lipid storage, maintenance of location, positive regulation of lipid storage, and regulation of cardiac muscle contraction by adjusting sequestered calcium ion and release regulation of lipid storage (**Figure 5D**).

### Validation of the expression and prognostic value of TRIM8

**Figure 6:**
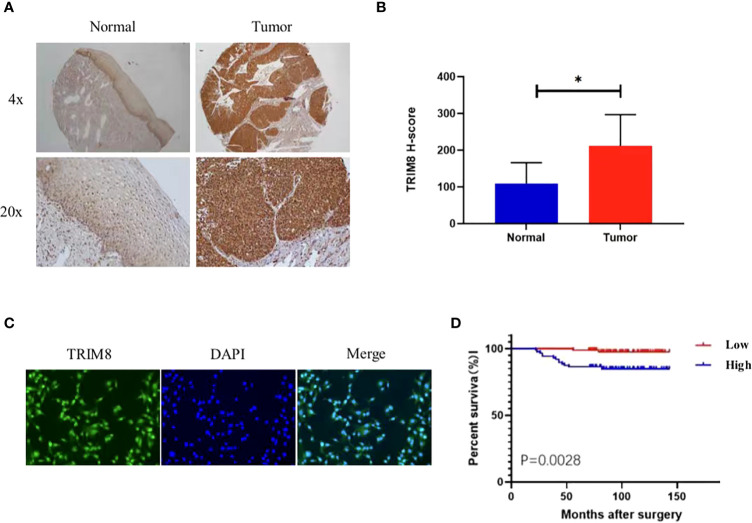
Expression and prognosis of TRIM8 in cervical cancer. **(A)** The IHC showed that TRIM8 protein is highly expressed in tumor tissues. **(B)** Statistical results of IHC score of TRIM8. *p < 0.05. **(C)** Distribution of TRIM8 in cervical cancer cells. **(D)** Kaplan Meier curve analysis of patients with high and low expression levels of TRIM8.

Immunohistochemical staining was used to detect the expression of TRIM8 in 176 cervical cancer tissues and corresponding normal tissues in tissue microarray, which confirmed the higher TRIM8 expression in cervical cancer than that in corresponding normal tissues, as well as the presence of TRIM8 expression in both cytoplasm and nucleus (p<0.05) (**Figures 6A, B**). Besides, immunofluorescence reveals the superior TRIM8 expression in the nucleus compared with the cytoplasm (**Figure 6C**). The immunohistochemical staining score of TRIM8 in cervical cancers was divided into high and low groups by median method. Among 176 cervical cancer tissues, 88 cases had high expression of TRIM8, and 88 cases had low expression. The χ2 test was used to analyze the relationship between TRIM8 and the clinicopathological characteristics of cervical cancers, demonstrating the close relation between high TRIM8 and FIGO stage (p=0.012), tumor size (P<0.001), and lymph node metastasis (P=0.034). There was no correlation with other clinicopathological factors (such as age, pathological type, degree of differentiation, depth of interstitial infiltration, vascular infiltration, and postoperative adjuvant treatment) (**Table 1**). Kaplan Meier analysis was used to discover the relationship between TRIM8 expression and overall survival of patients, which confirmed that cervical cancers with high TRIM8 expression had a shorter overall survival, while those with low TRIM8 expression had a longer overall survival (p=0.0028) (**Figure 6D**). Accordingly, the association between the prognosis of cervical cancers and FIGO stage (P<0.001), depth of invasion (p=0.008), lymph node metastasis (p=0.006), postoperative adjuvant therapy (p=0.042), and TRIM8 expression (p=0.010) was validated. Multivariate Cox regression analysis found that lymph node metastasis (p=0.001) and TRIM8 expression (p=0.028) were independent prognostic factors for the survival of cervical cancers (**Table 2**).

**Table 1:** Relationship between TRIM8 and clinical characteristics of cervical cancers.

Characteristics	Group	Total	TRIM8		χ2 value	P value
			Low-exp	High-exp		
Age	≤55	89	51	38	3.841	0.050
	>55	87	37	50		
Pathological type	SCC	152	77	75	0.193	0.660
	No-SCC	24	11	13		
FIGO stage	I	143	78	65	6.303	0.012*
	II	33	10	23		
Degree of differentiation	I-II	95	44	51	1.121	0.290
	III	81	44	37		
Tumor size	≤4cm	134	78	56	15.13	<0.001***
	>4cm	42	10	32		
Depth of stromal invasion	≤1/2	126	67	59	1.788	0.181
	>1/2	50	21	29		
Vascular invasion	No	148	75	73	0.17	0.680
	Yes	28	13	15		
Lymph node metastasis	No	143	77	66	4.513	0.034*
	Yes	33	12	21		
Adjuvant therapy	No	57	33	24	2.717	0.437
	Chemotherapy	66	31	35		
	Radiotherapy	3	2	1		
	Chemoradiotherapy	50	22	28		

Annotations: *P < 0.05, ***P < 0.001.

**Table 2:** Univariate and multivariate analysis of overall survival in cervical cancers.

	Univariate		Multivariate	
	HR (95% CI)	P value	HR (95% CI)	P value
Age (≤55 vs >55)	1.582 (0.563-4.444)	0.384		0.841
Pathological type (SCC vs No-SCC)	1.221 (0.425-3.509)	0.710		0.193
FIGO stage (I vs II)	7.801 (2.733-21.942)	<0.001***	6.223 (2.193-17.658)	0.001**
Degree of differentiation (I-II vs III)	1.020 (0.370-2.813)	0.969		0.121
Tumor size (≤4cm vs >4cm)	1.156 (0.368-3.630)	0.804		0.136
Depth of stromal invasion (≤1/2 vs >1/2)	4.016 (1.429-11.286)	0.008**		0.788
Vascular invasion (No vs Yes)	1.365 (0.385-4.838)	0.630		0.170
Lymph node metastasis (No vs Yes)	4.165 (1.507-11.511)	0.006**		0.786
Adjuvant therapy (No vs Chemotherapy vs Radiotherapy vs Chemoradiotherapy)	1.542 (1.015-2.342)	0.042*		0.717
TRIM8 (High vs Low)	7.006 (1.581-31.051)	0.010*	5.366 (1.197-24.063)	0.028*

Annotations: *P < 0.05, **P < 0.01, ***P < 0.001.

### The malignant behaviors of TRIM8 in cervical cancer

**Figure 7:**
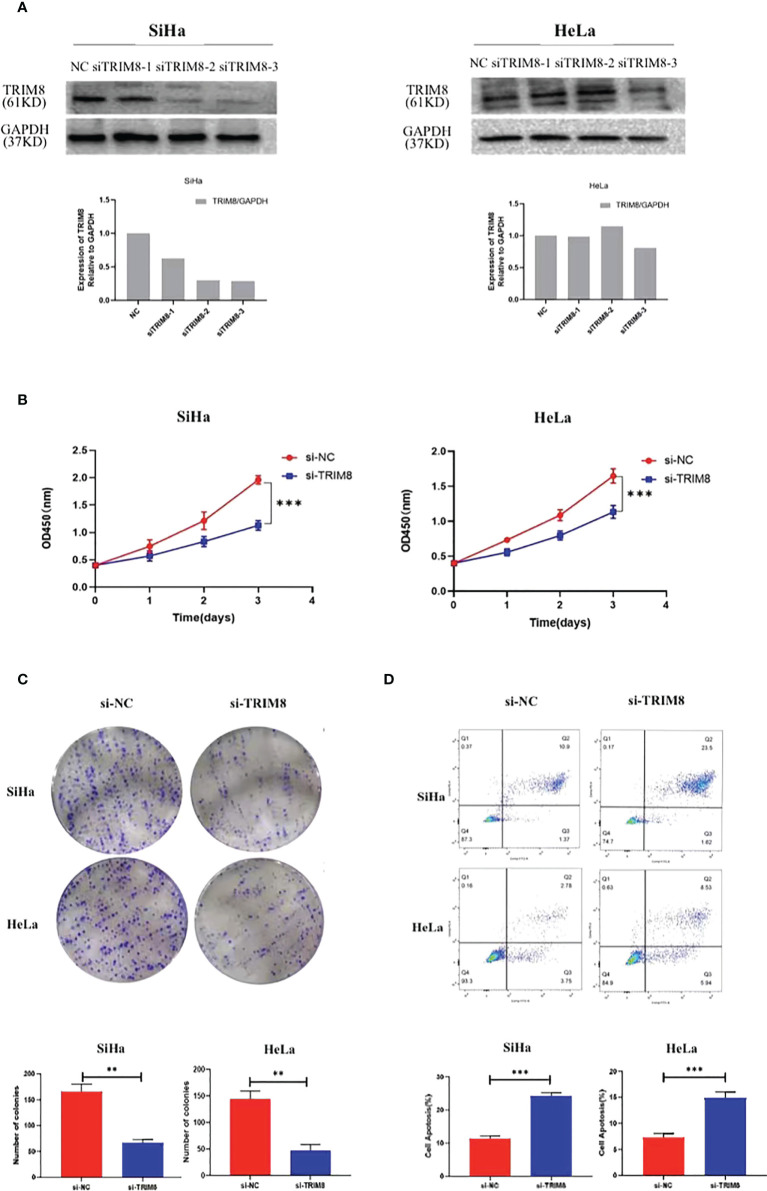
TRIM8 plays a role as an oncogene in cervical cancer. **(A)** Verification of interference efficiency of TRIM8 in SiHa and HeLa cells, respectively. The bottom figures are the corresponding quantization diagrams. **(B, C)** CCK-8 and clone formation assays showed that down regulation of TRIM8 expression inhibited the proliferation of SiHa and HeLa cells. **(D)** Flow cytometry indicated that the silence of TRIM8 increased the apoptosis ratio of SiHa cells and HeLa cells. **p < 0.01, ***p < 0.001.

To verify the involvement of TRIM8 in the tumorigenesis and development of cervical cancer, three siRNAs were designed to silence the TRIM8 expression in cervical cancer cells SiHa and HeLa. Immunoblot analysis was also used to confirm the knockout efficiency of TRIM8, which revealed that knocking out with siRNA-3 reduced the TRIM8 expression by more than half in two cells. Therefore, siRNA-3 was selected for subsequent cell function experiments (**Figure 7A**). The effect of TRIM8 on the proliferation of cells was detected by CCK‐8 kit and clone formation assay. Specifically, the CCK-8 kit experiment indicated that the decreased TRIM8 expression reduced the SiHa and HeLa proliferation activity (P<0.001) (**Figure 7B**), while the clone formation assay revealed that interference with TRIM8 inhibited the colony count of SiHa and HeLa cells (P< 0.01) (**Figure 7C**). The effect of TRIM8 on apoptosis of cervical cancer was detected by flow cytometry, which showed that compared with the control group, the silence of TRIM8 increased the apoptosis ratio of SiHa and HeLa cells (P< 0.001) (**Figure 7D**). Therefore, the TRIM8 gene was found to be involved in the proliferation of cervical cancer.

### Drug sensitivity analysis and drug prediction

**Figure 8:**
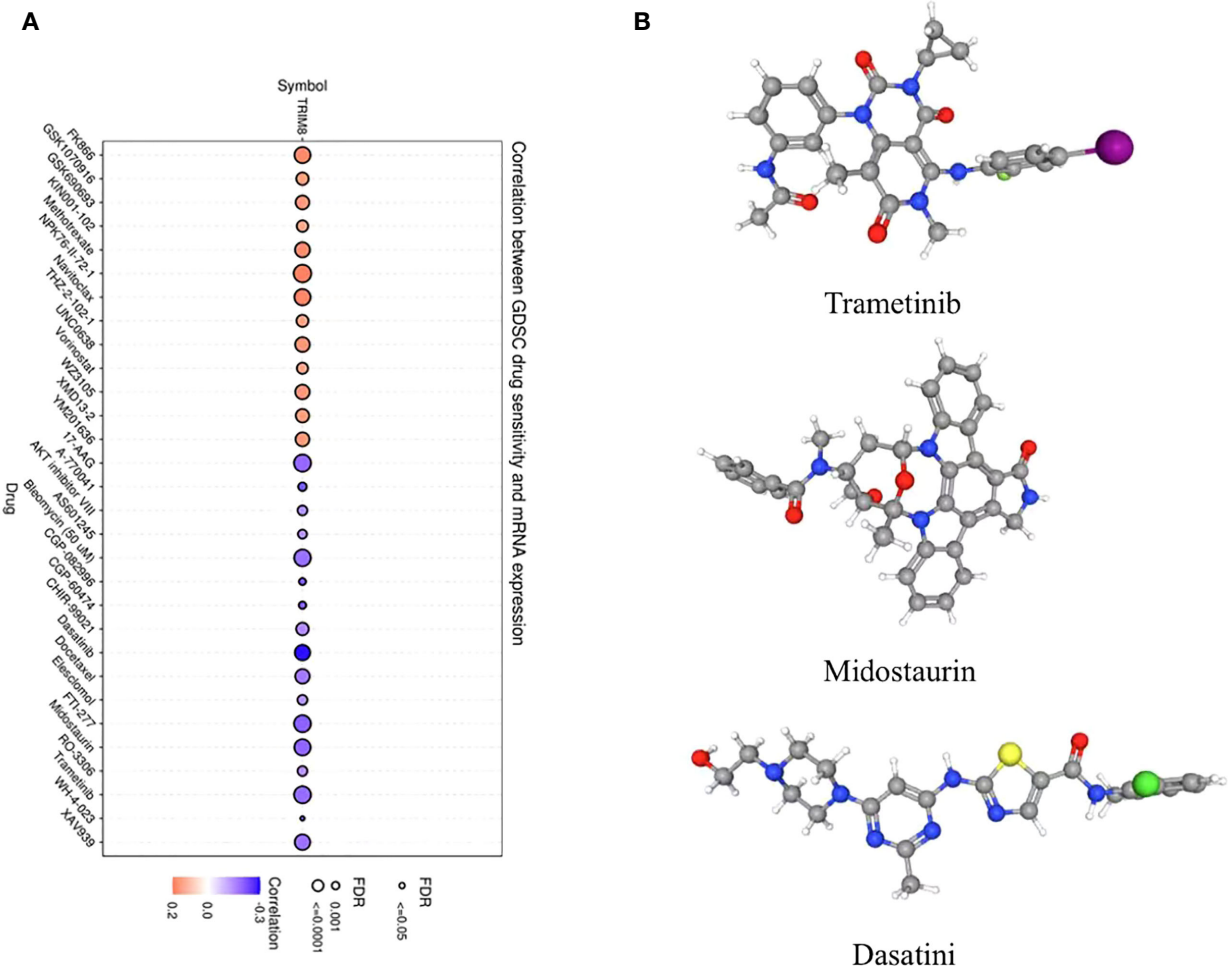
Analysis of potential drug sensitivity of TRIM8. **(A)** The Gene Set Cancer Analysis (GSCA). **(B)** Structure of potential targeted drugs, including trametinib, midostaurin, and dasatinib.

In order to explore the molecules that can be used as targeted drugs, the drug sensitivity of TRIM8 was analyzed. The top three drugs most related to TRIM8 were selected. Potential targeted drugs were also identified, including trametinib, midostaurin, and dasatinib, which could improve patient survival (**Figure 8A**). Based on Spearman correlation analysis, the structure of potentially targeted drugs was identified (**Figure 8B**).

### ssGSEA

**Figure 9:**
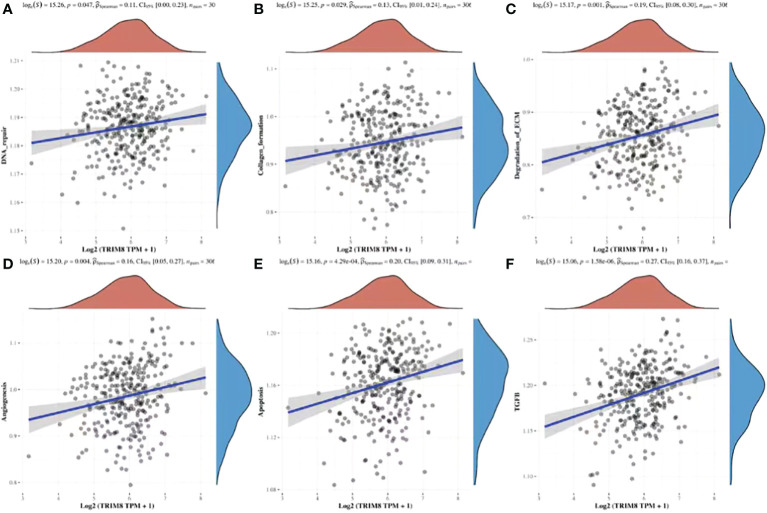
Spearman correlation analysis between TRIM8 and pathway score. **(A–F)** The signal pathway positively correlated with the high expression of TRIM8 was screened.

By utilizing TCGA database, TRIM8 was divided into high expression and low expression data sets according to the median value, and ssGSEA was employed to detect which pathways were enriched in TRIM8 high expression samples. The most important signal pathways were screened based on normalized enrichment score (NES). As shown in **Figure 9**, DNA repair (A), collagen formation (B), degradation of ECM (C), angiogenesis (D), apoptosis (E), and TGF-β (F) were enriched in cervical cancer samples with high TRIM8 expression.

### TRIM8 substrate and co-expressed miRNA analysis

**Figure 10:**
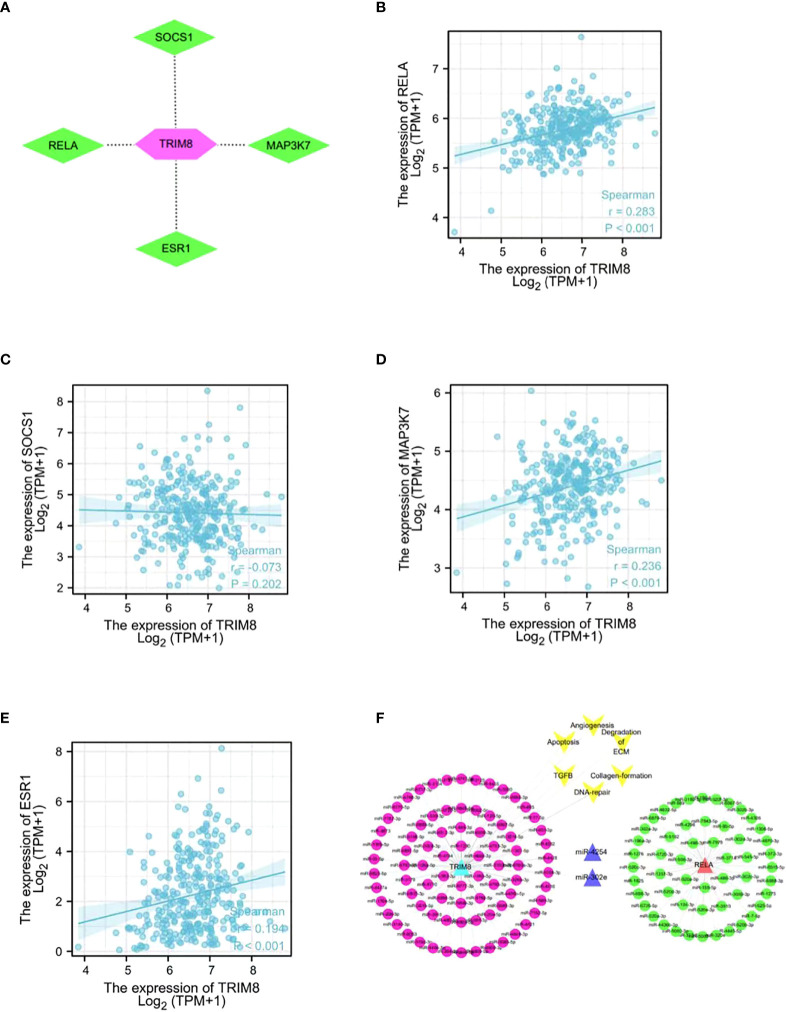
Network and function of TRIM8 and its substrate genes. **(A)** Network view of predicted E3-substrate interactions in UbiBrowser web services. In network view, the central node is TRIM8, and the surrounding nodes are the predicted substrates. **(B–E)** Correlation analysis of TRIM8 and four predictive substrates in cervical cancer. **(F)** Schematic diagram of interaction of co-expressed miRNA network of TRIM8 and RELA.

In order to identify the potential substrate of TRIM8, TRIM8, as the ligase substrate of E3, was queried in the web tool of UbiBrowser to identify the ubiquitinated protein target of TRIM8 that is overexpressed or underexpressed in the progression of cervical cancer. **Figure 10A** shows four substrates, including three known substrates SOCS1, MAP3K7, and ESR1, and a predicted substrate RELA. Then, a correlation analysis was performed, revealing the positive relation between TRIM8 expression and the expression of four substrates, among which RELA was the most correlated (R=0.36, p<0.001) (**Figures 10B–E**). Common miRNAs based on TRIM8 and RELA were sought to explain possible signal pathways, finding a total of two commonly predicted miRNAs, miR-4254 and miR-302e (**Figure 10F**).

## Discussion

Cervical cancer, as one of the most common malignant tumors suffered by women, exhibits complex and multifaceted pathogenesis (19). Despite the advance in the prevention and treatment of cervical cancer, the molecular mechanism of its progression remains unclear. Thanks to the progress in bioinformatics and the development of high-throughput technology, an increasing number of researchers identify new biomarkers of cancer progression by analyzing microarray datasets in public databases and mining effective information, which highlights the need to assess the gene expression profile of cervical cancer, thus better evaluating the clinical outcomes.

The paper first analyzed the raw data in GSE39001, identified 2,859 DEGs, and performed pathway enrichment analysis on the DEGs. Then, a WGCNA co-expression network was constructed to classify the DEGs into different modules. WGCNA is a systematic method that divides genes with similar expression patterns into the same modules, and further explores the correlation between modules and clinical traits (20, 21). The ubiquitin-proteasome system is critical for the biological process regulation of tumor cells (22). Dysregulated ubiquitination and ubiquitination are closely related to the progress of cervical cancer (23, 24). In this study, ubiquitination-related genes were extracted from the top two key modules, and a PPI network was created to identify hub genes, whose expression correlation and potential biological functions were analyzed later.

Cervical cancer is a major public health problem worldwide. Molecular, epidemiological, and clinical investigations have identified HPV as the main cause (25). Autophagy is a highly conserved multi-step lysosomal degradation process, in which cellular components are limited to autophagosomes, and then fuse with lysosomes to degrade chelated contents (18). The close relationship between autophagy and tumor progression is confirmed. Peng et al. proved that autophagy can maintain the dryness of ovarian cancer stem cells through FOXA2, thus promoting the progression of ovarian cancer (26). In addition, autophagy is considered to be the critical cause of chemical resistance in colorectal cancer treatment, which facilitates the development of anticancer therapies targeting autophagy pathway (27). Autophagy plays a key role in mediating tumor virus infection and tumorigenesis, and restoring autophagy response, which greatly inhibits HPV-mediated diseases, especially cervical cancer (28). Recent studies have confirmed that TRIM65-mediated p53 ubiquitination and degradation can directly inhibit apoptosis, reduce autophagy flux through the classical mTOR signaling pathway, downregulate autophagy related apoptosis, and ultimately promote the occurrence and development of cervical cancer (29). Therefore, we extracted autophagy-related genes from 44 hub genes for analysis, and found only one qualified gene TRIM8. The ROC curve confirms the relevance of TRIM8 to clinical practice. Subsequently, tumor samples were divided into high and low TRIM8 expression groups to better study TRIM8 related genes and molecular characteristics. The CSCs score showed the superior characteristics of tumor stem cells in TRIM8 high expression group compared with normal control group. According to mutation analysis, frequent mutation were observed among EP300, PRUNE2, STAB1 etc. in high TRIM8 expression group, while among EP300, DMXL2, PLXNA1 etc. in low expression group.

TRIM8, as a member of the tripartite motif (TRIM) protein family, has a common domain: a RING-finger domain, one or two B-box zinc finger domains, a double helix domain, and a C-terminal variable domain (30). Most TRIM family proteins exhibit E3 ubiquitin ligase activity, and participate in the ubiquitination modification of a variety of substrates. There are more than 70 known TRIM proteins in humans and mice. Given the differences in the C-terminal domains of TRIM family proteins, TRIM proteins are divided into I to XI subfamilies (31–33), which are vital for inflammation, immunity, tumor, signal transduction, cell proliferation, and apoptosis (34–36). TRIM8, originally found in glioblastoma, is also a designated glioblastoma expressed RING finger protein (GERP) (37), which is widely distributed and expressed in human tissues and tumors. Research has found that the RING finger domain stabilizes and activates P53, degrades MDM2, and leads to the instability of Δ DNp63α, which regulates cell proliferation (38, 39), while the B-box domain and coiled-coil domain interact with tumor suppressor gene SOCS1 (40). The helical domain is necessary for homodimerization (41), and the deletion of the C-terminal domain leads to protein localization errors (42).

TRIM8, which performs both carcinogenic and antitumor activities in tumors, acts differently in tumors. Studies have shown that TRIM8 overexpression inhibits the proliferation and invasion of laryngeal squamous cell carcinoma and breast cancer (43, 44). Caratozzolo et al. found that the decrease of TRIM8 expression was related to the malignant transformation of renal cell carcinoma cells, and that TRIM8 promoted the sensitivity of clear renal cell carcinoma cells to chemotherapy by restoring the activity of P53 (45, 46). Micale et al. demonstrated that the low expression of TRIM8 was related to the poor clinical prognosis of grade III neuroglioma, and affected cell proliferation (47). Despite the recognized identity of TRIM8 as an inhibitor gene of tumors, studies have also reported that TRIM8, as an oncogene, facilitates the occurrence and development of tumors. TRIM8 regulates the clone formation and migration ability of cancer cells by NF-κB pathway. Interference with TRIM8 in MCF7 and HEK293 will significantly reduce the proliferation and clonogenesis ability of cells (42). Therefore, TRIM8, as an oncogene, induces NF-κB pathway by promoting TNFα, which drives cell proliferation and migration. Research has reported that TRIM8 accelerates the occurrence of lysosomal organisms and autophagy flux under stress conditions, promotes the cleavage of c-caspase subunits, and enhances cell viability. TRIM8 is also proved to facilitate the stability of XIAP, and activate NF-κB. All these support the involvement of gene expression in autophagy and cell proliferation. In addition, TRIM8 mediates stable XIAP, and prevents the activation of caspase-3, thereby inhibiting cell apoptosis, and promoting cancer (48). Tomar et al. referred that the TRIM8 that enters cytoplasm from nucleus stimulated by TNFα plays a carcinogenic role in the nucleus and cytoplasm, and promotes cell proliferation and migration. They also mentioned that the silence of TRIM8 inhibits the activity of NF-κB (p65) in the cervical cancer cell HeLa (42), however, they failed to further explore the significance of TRIM8 in cervical cancer. Therefore, this study aims to clarify the potential role of TRIM8 in cervical cancer.

This study explored the significance of TRIM8 in cervical cancer through tissue samples and cell lines. IHC staining was first performed on the cancer and corresponding normal tissues of 176 patients with cervical cancer in tissue microarray, and TRIM8 expression in the nucleus and cytoplasm was confirmed, revealing the higher expression in cervical cancer than that in corresponding normal tissues. Then, the expression of TRIM8 was statistically analyzed with the clinicopathological characteristics of patients, which confirmed the association between TRIM8 expression and tumor size, FIGO stage, and lymph node metastasis. Subsequently, Kaplan Meier survival analysis found that patients with high expression of TRIM8 had shorter overall survival and worse prognosis. Finally, the combination of univariate and multivariate analysis showed that TRIM8 expression could be used as an independent prognostic factor for patients with cervical cancer. The above suggests the potential relation between TRIM8 and the occurrence and development of cervical cancer, its role as an index to judge the prognosis of cervical cancers, as well as its participation in the development of cervical cancer by affecting the proliferation. Therefore, we studied the effect of TRIM8 on the proliferation of cervical cancer cells in SiHa and HeLa by CCK8 and clone formation experiment. It was found that the interference of TRIM8 significantly reduced the proliferation, and increased the apoptosis of cervical cancer cells, which shared previous research results (36, 42). The drug sensitivity analysis indicated that small molecule drugs have potential clinical value in improving the survival outcome. Given the role of TRIM8 as an E3 ubiquitin ligase, its possible substrates and miRNAs regulated by upstream were predicted. In terms of mechanism, the possible signal pathways involved were listed.

## Conclusion

These findings are the original to report the expression and function of TRIM8 in cervical cancer. The study elucidates the function and mechanism of TRIM8 from the perspective of multiomics combined with the molecular information in the public databases, and the results provide novel insights into the comprehension on the pathophysiology of cervical cancer, and the development of therapeutic targets. This study, however, is limited in fully describing the effect of drug resistance and mutation characteristics, as only the expression and biological function of TRIM8 have been verified. Therefore, more efforts are required on the mechanism to figure out the way that TRIM8 affects cell proliferation and migration.

## Data availability statement

Publicly available datasets were analyzed in this study. This data can be found here: https://www.ncbi.nlm.nih.gov/geo/geo2r/?acc=GSE39001.

## Author contributions

AH and CP conceived and designed the study. YD, YY, CO, and QH performed the experiments. LZ, HQ and MT analyzed the data. LZ and AH wrote the manuscript. All authors have read and approved this manuscript.

## Funding

This work was supported by Nantong Science and Technology Bureau (grant no. JC2020025), by the Health Committee of Nantong (grant no. MB2020021, MA2021022), and by Clinical Medicine Special Program from Nantong University (grant no. 2019JY017).

## Acknowledgments

We would like to thank the GEO project for its valuable contributions to cancer research. We would like to thank the patients and their families and all the investigators, including the physicians, nurses and laboratory technicians in this study.

## Conflict of interest

The authors declare that the research was conducted in the absence of any commercial or financial relationships that could be construed as a potential conflict of interest.

## Publisher’s note

All claims expressed in this article are solely those of the authors and do not necessarily represent those of their affiliated organizations, or those of the publisher, the editors and the reviewers. Any product that may be evaluated in this article, or claim that may be made by its manufacturer, is not guaranteed or endorsed by the publisher.
