# Supplementary material for: Identification and validation of novel biomarker TRIM8 related to cervical cancer
Source: Front Oncol. 2022 Oct 24;12:1002040. doi: 10.3389/fonc.2022.1002040 (PMC9638460; doi:10.3389/fonc.2022.1002040)
Supplement: Supplementary file 1 [file Table_1.docx]

**Supplementary Table 1 Identification of DEGs in GSE39001**

| DEGs | Gene Name |
| --- | --- |
| Upregulated | SCGB1D2 CFD APOD SFRP4 CRNN PTCH1 SCGB2A1 WISP2 CXCL14 CRISP3 MAL MSX1 ESR1 SCGB2A2 CRCT1 TFF3 PTGDS FHL1 LOC100506718///FLRT2 DEFB1 SPINK5 PCP4 NDN SFRP1 ID4 IGF1 KRT13 DKK3 CYR61 PDGFRA OSR2 SPRR3 ALOX12 ANK2 PPP1R3C BCHE ABCA8 MYLK SRPX NR2F1 ACTG1P4///AMY2B///AMY2A///AMY1C///AMY1B///AMY1A LDB2 EDN3 MPPED2 COX7A1 RAI2 LOC100506558///MATN2 TGFBR3 FCGBP FBN1 ITM2A ADRA2A LRRC17 LTF SPARCL1 NDP HTR2B CYP2B7P///CYP2B6 CILP FOS GJA1 CYP1B1 IL1R1 LTBP1 SNAI2 NAP1L3 EDNRA DUSP1 PTGS2 DKK1 EXOSC7///CLEC3B CTSK FOSB PROM1 IGFBP6 JAM2 KCNJ15 GNG11 KRT1 ISLR FXYD1 SLC24A3 MITF LOC101928635///ALDH1A2 CRYAB COL16A1 ADAMTS1 PDGFD SSPN SERPINF1 CLU GRIA2 DIO2 ECM2 NCAM1 PLPP3 C4BPA S100A4 SOCS2 SCNN1B SLPI FABP4 TRH SLIT2 LEPROT///LEPR SCEL EGR1 TNS1 LOC101926921///DAB2 SLC18A2 FZD10 SGCE PRSS23 SORD NOVA1 GHR RUNX1T1 ADAMTS5 PCOLCE HP TGM2 GSTM5 MMP2 CDC42EP3 RHOB PLPP1 TUBA1A FGF13 PECAM1 CCL15-CCL14///CCL14 TGFB1I1 SORL1 IRS1 FAM107A EFEMP2 FBLN1 NT5E EMP1 GATA2 CIRBP PCSK5 FRZB CLDN10 ADRB2 HEPH PMP22 ALDH1A3 IVL PAM LMOD1 DKK2 SEPP1 RBPMS FGFR1 ASPN TCN1 ABCG2 LDOC1 FGF9 ALPP PRR16 IL20RA LOC101928916///NNMT KCNMA1 CX3CR1 BNIP3 DIO3 PKIG MAOB FMOD KIT KAT2B TCEAL1 SOX17 COLEC12 SLC39A8 THBD DACH1 RECK MINOS1-NBL1///NBL1 SCUBE2 EPHB6 ADIRF HPGD FOLR1 DCLK1 FBXL7 RGS1 PTGER2 GSTA4 RGS2 CHST7 MXRA7 CITED2 CHN2 BDKRB2 PLTP AQP1 INS-IGF2///IGF2 ACTA2 LAMA2 CTGF COL1A2 PEBP1 EDNRB NELL2 PTGIS STC1 COL3A1 IL33 THBS2 CRTAP F10 SATB1 ECI2 KLHL3 ME1 DPP4 KLF4 LHFP WFDC1 SNCAIP LAMB2 ZBTB16 CNN1 COL6A3 BCL2 ATP1A2 UPK1A PDGFRL MAF BBOX1 KLK11 HOXA10 RARRES2 CDA REV3L RRAGD KLK12 ENTPD3 PCDHGA1///PCDHGA2///PCDHGA3///PCDHGA4///PCDHGA5///PCDHGA6///PCDHGA7///PCDHGA9///PCDHGA10///PCDHGA11///PCDHGB1///PCDHGB2///PCDHGB3///PCDHGB5///PCDHGB6///PCDHGB7///PCDHGC4///PCDHGC5///PCDHGA12///PCDHGA8///PCDHGB4///PCDHGC3 PRNP ARHGAP6 DPT CFTR TST TPST1 ECM1 CH25H SALL1 RCAN1 IRS2 TACC1 STX18 SLC5A1 HTRA1 LAMC3 WFS1 NUCB2 SLURP1 ENDOU PDLIM2 GAS6 PDGFRB CACNA1D DSG1 MRC2 ACAT1 WASF3 VIM NSG1 PLAGL1 PENK ZFPM2 RCBTB2 CPA3 GAS7 FYN QPRT RCAN2 SAA2-SAA4///SAA2///SAA1 VCAN NRCAM GPX3 SMARCD3 ALDH6A1 TGFB3 CFH GEM ZEB2 IL1R2 ACPP FBLN2 CAV1 NR3C2 RAPGEF3 CDO1 PDE2A MAGI2 RGS5 PRKAR2B SPOCK1 ASPA ADGRL4 TCF12 SNORA11E///SNORA11D///MAGED4///MAGED4B EFS ACKR1 GREM1 NFATC4 COL5A1 NME5 SULT2B1 DYNLT3 SLC1A1 CCND2 FXYD6 SNURF///SNRPN TCF4 FOXF1 SLCO1B3 BACE1 EPHA3 PPL SYNGR1 RGN GADD45B MAP1A PDS5B DTWD1 GRK5 CLIC5 MOAP1 KAT6B CAMLG CCND1 LOC100130872///SPON2 CD248 CXCL12 HNMT ISL1 A2M CD34 MYL9 TNXB///TNXA PLLP COL18A1 CCNG2 AEBP1 IGFBP4 BDH2 GADD45G DACT1 SOD3 LAMA4 SSBP2 LAMP5 RAB2A SORBS1 CLDN5 DPYSL3 WWOX VAT1 PEMT ITGBL1 KLF2 SLCO2A1 HSPB2 DUSP6 PHYH MEIS1 TPM2 TSPAN7 GYS2 IL19 RPL31 SLC2A10 OMD SST DLC1 MAN1C1 WT1 LOC100653057///CES1 EYA1 CTSG PDE4A F13A1 TLE1 MED16 TEK SERPINA5 SLC15A2 TRHDE MAGEL2 EIF4B PDE1A SESN1 ARL6IP5 DIAPH2 FZD7 NOTCH2 RHOQ ACAA1 PKD2 SNORD23///GLTSCR2 SMARCA1 LMO2 GSTM2 NISCH SLC24A1 RAI14 HSD17B6 LZTFL1 MAPK10 SAA2-SAA4///SAA4 CLEC11A GSN SLC22A17 SCN1B REPS2 ATP7A STAC DKK3 FJX1 CDH2 FZD1 PIP CAPN6 EPB41L2 ASMTL AOC3 NCOA1 ALOX12B EGR3 RPL15 TGM3 PARVA MAFF ZNF580 ADARB1 TFCP2L1 KRT2 LTBP2 FZD4 TGFBR2 LOC101060817///GCSH IL11RA ME3 ZNF185 CAT PTPRN2 TRPS1 ERLIN2 CRYL1 ROBO1 GLT8D2 CES2 EGR2 CTSF SHC3 AP1S2 CLDN8 AR ITGB5 ADRA2C FOXL2 SLC4A7 ACSL4 SNAPC3 IGFBP7 KHDRBS3 DUSP14 CDH13 WIPF1 PLA2G4C FAM127A CDH12 PLAG1 C4orf46///TOMM7 KCNJ8 CELF2 EREG TRPC1 DHRS9 PTH1R CUL5 PIM1 DEGS1 ACOX2 TCF7L1 FAM8A1 RBFOX2 TK2 C7 ZNF83 MAN1A1 SEMA5A MEF2C COL5A3 VWA5A ADD1 PTK6 SPINK2 NBEA RAB31 COX11 AMT APBA2 GSTM1 LINC00312 TBL1X DPYSL2 EFNB2 RAMP1 NPY1R PFDN5 EGFL7 CKB P2RX5-TAX1BP3///TAX1BP3 HPGDS FBXO3 CA10 BTG2 ESRRG ATP5L ORAI3 NAP1L2 RBM6 ITPR2 RERE CD36 FAM117A JUN MNT PER2 CPQ PHF1 JRKL CCNG1 LRP3 KLK7 REN OPHN1 PGR PCDH9 NEFM PRKD1 KLF6 ZKSCAN7 SLC27A6 PCCA CAPN3 WNT4 B4GAT1 MXD1 ITSN1 EMP3 CCNI PCDHA1///PCDHA2///PCDHA3///PCDHA4///PCDHA5///PCDHA6///PCDHA7///PCDHA8///PCDHA10///PCDHA11///PCDHA12///PCDHA13///PCDHAC1///PCDHAC2///PCDHA9 PTPRM PCDH17 AMFR FOXA2 COL13A1 DLX5 INSR AFDN-AS1 CTTN ARG2 SLIT3 TERF2IP CLDN17 EFNB3 TIE1 VPS4B RPRM PCMT1 PBXIP1 ACADL RPS25 ARL4D SLC16A2 ENPP1 GLT8D1 BMPR1A PPP1CB ITPK1 PGF RBMS1 PALM ITPR1 SEMA3G TSPYL2 CMA1 GRIK2 PROS1 AASDHPPT EML1 HEY2 CD83 ZFP36 KCTD7///RABGEF1 PRELP RASL12 VWF EMCN ATXN1 ITGA10 NMT2 PLCB4 TCEA2 FLT1 KIF5C RPL36 SH3BP5 ASAH1 PRRG1 ZNF302 ANXA6 RELN COG5 MME HSD17B14 KCNAB1 CD69 PCOLCE2 LHPP NPR2 GYPC DPP6 AMPH GSTA1 NSMAF ELF2 VEGFC AGT PAEP RGS17 CYP39A1 INPP5A SLC7A8 CHAD ADCY9 PRCP PKP4 HDAC4 HBP1 CYP27A1 ZNF135 CRISP2 TRIM8 PPP1R12A STXBP1 ADAMTS9 SPHAR///RAB4A NPAS3 NDUFB8 PDPN PCDH7 XPA FAM65B ZNF85 TFAP2C CHST4 NME4 PEX7 COL4A3BP BMP4 PPP2R5C TRMT1L APPBP2 CYTL1 GAB2 CACNA1G ZC4H2 FLRT1 EVI5 ALDH9A1 FYCO1 MZF1 NR3C1 SH3GL2 PTEN DDAH2 VPS51 AGTR1 SELP SMPD1 CCL21 CTNS TP53AIP1 AMPD2 B4GALT1 VIPR2 ZNF189 CCDC85B LOC100996693///SPEG FOXO3 ZNF137P LEFTY1///LEFTY2 REM1 TBX2 UBE2B RGS6 HIGD1B HRC FEZ2 UBE2E1 LYVE1 PIP4K2B C3orf18 BST1 POU6F1 TNS1 EPB41L3 TNFSF12-TNFSF13///TNFSF12 CALD1 TBCD MUT CRBN RAMP2 THRA DLK1 CHKB AGA RANBP9 SEC31A TBXA2R PEX3 IDE SCAMP1 GJA4 SUPT3H LRP1 SOX13 FOXO1 PURA C1orf21 PMM1 ABCB4///ABCB1 HDC AKTIP ROR2 MPC1 EI24 WIF1 LINC00094 RPL36AL GABBR1 RNF139 AUH PRDM2 ATP5I TNKS SUOX ITGA7 ZNF14 CD1C ATP2B2 PEX12 LIPT1 TMEM8B RTN1 VPS33B CYB5R3 PAK3 ASIC2 MIR4800///MXD4 C6 MEOX2 DMD AMOTL2 PRPH2 TBL2 PTGER1 MAP4 PRMT2 FDX1 P3H4 STMN2 RNF39 SPACA9 ARCN1 IFT88 KRT76 GNG7 EVC IGFBP5 SLC26A10 KLKB1 UQCRB CBFA2T3 NAT6 TLK1 CA4 PAFAH1B1 TOM1 TAC1 PRRX2 PRL POLR2A EZH1 ZBTB22 LEPROT NFAT5 C21orf33 GUCY1A2 CDH5 FANCF UBR5 NRN1 RPL32 LOC102724229///RASA4B///RASA4 PLA2G4B///JMJD7-PLA2G4B SARAF FGF2 AMPD1 NRXN3 DNAI2 PPARGC1A EIF3E GRIA3 HIBCH CHGA PCF11 CPQ GTF3C1 ATP1B2 GTF2H5 MYBPC1 ZMYND11 ROR1 RARA FOXC1 CHST12 LOC149684///BPI CREBL2 MLYCD FABP6 CSF3 SEC24D MAGI1 NELL1 HPSE2 OR2C1 HSPB7 CGRRF1 RAB5A NR5A2 DPEP1 STX8 TAS2R1 CTDSP1 CLOCK GGT5 OGG1 MIR6883///PER1 DNAH9 CD177 DES TCIRG1 ZFYVE9 RAMP3 GFPT2 SNORD18C///SNORD18B///SNORD18A///SNORD16///RPL4 KDR CHST10 SERPINA6 ZNF134 NXF3 KLHDC2 SYNCRIP TXN2 POLR1E LSAMP C21orf2 LAPTM4A GPR22 PGRMC2 OCA2 NRF1 AFF1 EIF4E AMD1 LY6G6C GOLGA4 FOLH1 MAN1A2 CSNK2A2 PLCD1 SETMAR PCDH12 TRPV2 PEX5 AASS RPL31 RAD52 KIF5A CYP2A6 LRRC32 GOLGA3 RPL27 RPL34 ERCC5 CDIPT SIL1 UQCR11 CNPPD1 SACS RAB6B TPSG1 ITIH3 CHD2 COL9A1 SRR PLN ITGA9 TOP2B PCDHGA9 IDS FAM102A GRIN1 SNORA41///EEF1B2 SLC6A3 EDA NACA FAU ZNF559-ZNF177///ZNF177 GRHPR TSN GABRG2 TDRD1 RPS6KA2 CSAD NME3 CLEC1A LDLRAD4 PRPH RPL10A DOK1 HAAO G6PC2 ELSPBP1 CACNB1 TCTA CACNA1H CKMT2 TSNAX-DISC1///DISC1 CACNA2D2 CNR2 LINC00597 FLNC ATP6V1B1 NPY POLI NLRP1 MAP2K4 TSPAN32 NOL4 HIST1H3E TMED3 LOC101930578///MRPS31P5///THSD1 NR1D1///THRA AKAP8 USP29 PPP3CB SCN5A KCNMB4 MMP24-AS1 COX7C BSN IGBP1 ACAA1 FAH TAL1 MMRN1 EDF1 SYN2 PPP1R1A KCND2 TRDMT1 CNR1 ZDHHC3 SLC6A2 ERAP1 EPS15 NTRK2 LOC100508408///SNORD14B///RPS13 GRM2 GDF5 SHBG MMP19 NLGN3 KLF9 ADAMTS2 PHC1 FAM3A TIMM22 TMEM256-PLSCR3///PLSCR3 DDX17 FHIT ACVRL1 C1QTNF1 PCBP3 CDRT1 CTNNBIP1 GLI1 MAP4 PRRG3 NTF3 GTPBP1 TAF1C MFGE8 NEBL ADAMTS13 KCNA10 MORF4L2 GRK4 GNA12 PRDM12 SCRG1 RPS27A SNORD45C///SNORD45A///SNORD45B///RABGGTB TIMM9 NDST2 CCL1 RPS28 TRIM6-TRIM34///TRIM34 CLUL1 GSTA3 PDSS2 NOX5 LOR HBZ CRB1 ATP12A TMED2 KRT36 HHLA1 TROVE2 THRB TNFRSF8 HMG20A PHKA2 DCT CHST8 EPOR UROD FCN3 MYOZ1 GRIK1 SCN2B RPL21P28///SNORA27///SNORD102///RPL21 E2F4 SLC31A1 MEOX1 CORIN SEC11A LRRC2 S1PR1 PRKACA EIF3H SNORA52///RPLP2 SHOC2 MCHR1 IGF1R SORBS3 TSGA10 CYP46A1 SEPT5-GP1BB///SEPT5///GP1BB UBXN1 PPP2R1B SLC17A1 SHPK///TRPV1 SLC38A3 OR6A2 FGF1 KCNK7 NEU3 CNNM1 TUBG2 DCP1A PARVB CRCP XPC CER1 WDR4 ZIC3 LOC101930075///PKD1 VGLL3 CDKL3 ARL2 PTPRG KLHL20 RXRG APBB1 ITIH4 DVL2 SPTB MYOM2 MYOT LOC100653049///KRT34 SLC5A5 CLEC10A PRR3 LAMP1 ZFP37 NRG2 BICD1 DKK4 MLH3 CDIP1 STK32B FOLR2 CRMP1 KRTAP5-8 CRYZL1 SERPINF2 CTAG1A///CTAG1B CTRB2///CTRB1 MAFK ZNF350 PPP5C SCN3B WNT2B POMT1 BACH2 LYL1 FSTL3 RNASE3 COPS7A OR1A1 APLNR OR1G1 PRDM16 LINC01565 MYH13 RHBG SFTPB SLC43A1 GPR6 LZTR1 ADPRH MTMR3 CA8 NTRK3 SMAD9 SYT1 CNGA1 DRP2 ASIC1 TUBB4A SNCG RPL37A MLN ZNF277 MYL3 TIAL1 T PDCL WASF1 CSRNP2 KRT33B TMPRSS15 HIST1H4I DLGAP2 SYN3 MMP27 NR4A1 OTC ABCG5 KLF8 CABYR MAPK11 CYP2A7 CYP17A1 RPS29 WNT8B CA5A MAGEC2 FGB CBLN1 FHOD1 FHL5 NUMA1 ATP4A CHRNA1 GUCY2F CRHBP EIF4EBP2 COX7A2L ANO2 IL36A NPY5R PLIN1 SKP1 RWDD2B CADPS SSX3 SEC61B BRE WRN TSR3 GSTT2 SYNC PNLIPRP1 OPRM1 KIR2DS1 SLC17A4 NAALADL1 MAS1 GCAT KALRN AZI2 BHMT IL9R KRT37 MIR6831///APBB3 C1orf61 HAS1 PCDH11Y///PCDH11X NEUROD6 GLI2 SDPR RNASEL TMEM257 BRINP1 POLL GUCY2D SLCO5A1 OR7C2 CHRNG OPCML UMOD NUDT2 GRM6 NGF NBEAL1///RPL12 FGF18 HAP1 MCC HUNK SLC6A5 FCN2 LPAR1 BHMT2 ZRSR1///SRP19 KCNJ1 APOH STAG3L4 PARK2 TRAF6 ARG1 UBE2D4 NYX ADGRB3 RRN3P1///RRN3P2///RRN3 OPN1SW SMR3A///SMR3B AKAP6 AOC2 KRT32 SLC4A1 CYP11A1 SLC22A8 AIPL1 TAAR3 MYH7B LIFR HSPB3 LOC643733 NOS3 SCAND2P NGB INPP5E FOXJ2 CDON FEV PPP2R5D LINC01558 CD40LG CHRM5 MPDZ MMP24-AS1 DEFA5 KALRN ACKR2 CYP3A4 TAS2R8 IKZF2 TUSC2 TRO PTPRJ GAGE1 CNTNAP1 USP20 HAL GJC2 MAGEA9B///MAGEA9 CHRNA4 RPS12 CXXC4 CSN1S1 RPS17 DGKG C5AR2 CSNK1A1 PCDHA2///PCDHA3 DPYS SPP2 HCFC2 OMP ACTR3B TYRP1 NEU2 FHL3 SNX2 DIRAS3 SGCA PPIL2 PVALB MAMLD1 ODF1 CCL23 FGA REG3A SLC6A4 SNPH TAS2R16 BMX SLC9A5 TCF21 STK19 PTGER3 VIP RGS12 SSUH2 OSBP ZNF254 PCDHGB5 S100A5 SLC6A9 OSM DCAF8 CISH ACSBG1 CASQ2 CRYGD MYBBP1A CYP3A4 MAP2K5 PMPCB DLG4 FUT5 TRPV5 LGALS14 RYR2 SERPINC1 TNFRSF4 PRM1 GRK2 UCP1 SCNN1G CNOT2 IKZF4 PPP3R1 TSHB GSK3A SHOX CHRNA10 MN1 PZP FN3K FSHR RPL9 SLC6A12 NXF2B///NXF2 ERBB4 ADAM12 RPS10 OR1F1 MID2 UBAP1 TFAP4 SSX2B PTGDR2 SCARF1 DPF1 CST2 RPS6KA6 MAPK4 SLC6A1 CTAGE1 APOC4-APOC2///APOC4///APOC2 RND2 NRL MVK CH17-360D5.1///NPY4R LHCGR CIITA FOXN2 PTGIR SFRP5 DNLZ///CARD9 ARSB MPPED1 CLCNKB///CLCNKA RTN2 RDH8 SLC18A3 MAP1B TTR CCR9 TAGLN3 CELF3 TNNI1 SLC12A3 AQP2 ZNHIT2 GPR15 IGHMBP2 OR1E2///OR1E1 FAXDC2 GABRA2 GC REG1B FZD3 ZNF335 B3GALT1 SLC2A11 MPZ HEYL FAIM2 IRF4 SPACA1 GLRA1 TADA2A GDF11 RASGRP2 APOB PRH1-PRR4///PRR4 SFTPC CACNA1I SLC6A7 PSG2 SNORA3A///RPL27A OR2S2 ANGPT4 SCN4A ANGPTL8 ST8SIA2 CLEC4E TDGF1P3///TDGF1 MC5R NAT2 CETN1 CHRM4 SLC22A6 RORB BCL9 TRHR LINC01587 USH2A PLP1 SRD5A2 SNORD73A///RPS3A TNP1 MRE11A GRM3 IL12A RPS10 IL5RA MMP16 RXFP3 MADD PAX7 CA5B XYLB TEX14 CCL27 OR2W1 TUB CASP2 CABP5 PTPN21 THPO HPS1 IL9 ANKH SMPX ZNF667 DDO CACNA1C RGS11 GLYAT TENM1 NR1H4 GPX5 MMP23A///MMP23B GRIN2B GRAP KL NR1I2 NR4A3 CD300C SHH MKRN3 LOC101930361///LOC101929970///CHRFAM7A///CHRNA7 FABP1 ACHE DLGAP1 CALCR TPH1 RPS23 MOG SLC2A4 PTGFR TPTE CDC14A GREM2 LY6H KEL PCSK2 RAPGEF4 TAAR2 ZNF197 ST8SIA1 KCNA4 RHAG BNIP1 SLC12A1 AVPR1A PMP2 CD226 ING4 PRLR LOC51145 PCDHGB6 |
| Downregulated | RRM2 NUSAP1 MMP12 TYMS CDC20 PRC1 UBD///GABBR1 KRT17///JUP RFC4 CDKN2A TOP2A CDKN3 ZWINT SYCP2 CKS2 CDK1 MMP1 S100P SMC4 GAPDH UBE2C MCM5 MCM2vHBA2///HBA1 KRT7 APOBEC3B CCNB2 GMNN CXCL10 PTTG1 AURKA MEST SLC35F6///CENPA CXCL9 TPX2 KIF20A BUB1B PLOD2 NEK2 HBA2///HBA1 MSLN UBE2S MAD2L1 LAMP3 RACGAP1 PLSCR1 GINS2 CDC7 HLA-DRA EZH2 PBK PCNA TOPBP1 HLTF SPP1 KIF4A NCAPG IL32 ISG15 SDC4 IFI27 SNORD14D///SNORD14C///HSPA8 CKS1B BIRC5 PSMB9 TTK GAPDH TACC3 NDC80 MCM6 SLC38A1 CXCL13 MUC1 IFI44L IFI30///PIK3R2 FEN1 KIF11 H2AFZ AIM2 ILF2 CA2 RFC3 ACTB RFC5 LAMB3 TIMELESS TK1 ATP2A2 CENPE RYR1 CXCL8 PDZK1IP1 F11R LSM4 IDH2 MX1 KRT19 MRPS12 IDO1 HN1 HOXC6 AURKA STIL ENO1 OAS3 CDH3 GGH LOC101060835///LOC100996809///HLA-DRB4///HLA-DRB3///HLA-DRB1///HLA-DQB1 PLAT PSMB8 DHX15 HPRT1 CCNA2 STMN1 CCT2 HSP90AB1 RAD51AP1 HNRNPF STAT1 OAS1 KPNA2 SCD RNASEH2A ID1 CXCL11 HBB PNP CDC25B DNMT1 GPR87 SMC1A NEFH MMP3 TRIP13 SELT CTSS MCM3 PSMC3 ITGB4 KRT8 LAPTM4B SLBP KIF15 TGFBI GMPS ASS1 RANBP1 TRIM29 TNFSF10 IRF1 POLE2 C1QB ACTB BARD1 EIF2S2 AP2S1 DSG2 EFNA1 DEK SNORA68///RPL18A PLP2 IRF7 P4HB GNB1 TAGLN2 HAT1 TUBG1 CCNE2 DDX39A SOX4 CDC45 RPN2 LIG1 PRIM1 IMPA2 PLAU BUB1 BTN3A2 POLE3 ATP1B1 NUP107 ISG20 NMI ELF3 CA9 BST2 CDKN2C ADAMDEC1 TAPBP MSH2 YWHAH HCAR3 ELF4 GCH1 RAN CTPS1 ACTL6A SHMT2 WHSC1 LAPTM5 LOC101060835///LOC100996809///HLA-DRB5///HLA-DRB4///HLA-DRB1///HLA-DQB1 SPAG5 GAPDH FZD6 MTHFD1 AP2M1 HMGB1 HSPA2 LAP3 SLC6A8 DDOST SNRPD1 FGFR3 USP1 KIF2C NCK1 CCNA2 SLC25A5 CTSC HLA-B CHI3L1 PSMB2 MICB STAT3 ITGB3BP MLF1 HIST1H1C LDHA CSNK1D PHLDA2 WARS CKMT1A///CKMT1B IFI44 ACOT7 HNRNPAB SDC1 GJB3 HSPA1L///HSPA1B///HSPA1A CD164 LAMC2 PPP1CA PSMA7 HLA-G CAPG PDXK PAFAH1B3 STAT1 IFI16 STAT1 C1QA RAD23A H2BFS VAMP8 LMNB1 HLA-DMA KIF22 PLAUR ITPR3 PSMD8 ERBB3 PGD ARPC5 RRM1 DCK CIB1 BLM MIR1282///HYPK///SERF2 NUP62 TNNT1 RC3H2 CLDND1 YWHAZ RUVBL1 CDK2 USP18 TYMP TMX1 PHB H1FX INHBA PSMD11 MTF2 SNRPB MKI67 BRCA1 FAT1 PSMD1 VDAC1 HNRNPU RAD21 LSR VRK1 KIF23 CYBA LSM2 NQO1 PDCD10 PRKCI ATP1A1 IL10RB SLC20A1 ALG6 PSMD12 ACTR2 MSH6 FCGR3B GDE1 PTPRF PAWR ZNF148 MYBL2 PKM ICAM1 HLA-DRB1 SLC16A1 CA12 MIR3658///UCK2 EIF4EBP1 PRDX1 XPNPEP1 RAD51 CEBPG HMMR ZNF207 HTATIP2 MTHFD2 CAPNS1 NIT2 MMP9 HLA-A DVL3 PFN1 ALOX5AP RPA3 MARCKS PDLIM5 DHCR24 SYNGR3 STAT1 DAZAP2 SLC2A1 BTG3 MGAT4B PSMB3 RBBP4 UBE2I RHOC PSMA1 HIST2H2AA4///HIST2H2AA3 PPM1G PFKP APOL1 GNB2 RALY ANXA3 GPSM2 TMPRSS4 NCBP1 EIF3B DBF4 CLIC1 EXO1 IRF9 PRMT1 PCCB PSAP EIF2AK1 HMG20B NME1 SCO2 TFDP2 CKLF-CMTM1///CKLF DHFR CYC1 HOXC10 ATP1B3 SPINT2 CDK4 TPD52 CACYBP PRKCSH TJP2 GSPT1 NUP155 AURKB MYO10 AHCY ASNA1 CHAF1B VEGFA ATG3 ABCA1 CHAF1A ENO2 ALDOA PIK3CA GART LOC101928634///SENP3-EIF4A1///SNORD10///SNORA48///SNORA67///EIF4A1 CD14 PFDN2 CDC25C FA2H ATP6V0B PPIH ADORA2B GZMB UMPS CCZ1B///CCZ1 PRPF4 PSMB1 FCER1G GDI2 PARP1 UBE2L3 PRKDC ASNS MIF HMGB3 SDF4 PRMT5 ARPC1B CASP8AP2 RPLP0 EEF1E1 ARPC1A PSMD2 ATG5 PIK3R3 PSMA5 CD74 PLEK2 ITGA3 TFAP2A MRPS7 VNN2 VAV3 SLAMF8 PSMA3 STIP1 IRAK1 HMGN2 CCL18 PCK2 PSMA2 HMGA1 FOXA1 MIR636///SRSF2 CDC6 CTNNA1 ABCG1 MAGOHB///MAGOH PSMD3 NCF2 PSMA4 SDHB PAX6 NUDT1 CTSA FBXO5 TROAP NPC1 PLPP2 CD58 COPA CORO1C ERGIC3 CXADR NSFL1C RAD54L CDC25A CLPTM1 LLGL2 RFX5 S100A11 CDC37 LGALS3BP LOC101060363///PPIA HSPA14 VAMP7 PRCC HNRNPA2B1 BANF1 MIS18A HERC5 SYNGR2 MDK SMS PDAP1 PDIA3 POLA1 TRAF4 BCL2A1 IFIT3 UQCRC1 TSTA3 OAS2 NFATC3 CD46 GPX1 CENPF TCP11 HDAC1 HSPE1 TPI1 CRIP2 TXNRD1 KDM6A MX2 PSMD14 SH3BGRL3 CELSR3 GATC///SRSF9 SLC7A5 SMC3 MIR6787///SLC16A3 NFKBIE HNRNPD MST1R ADSS GSTP1 YBX1 CDKN1A P2RX4 PXMP2 PSIP1 SEPW1 SP110 MAPRE1 TFDP1 BTN3A3 USP14 TEAD4 CBLC DTYMK U2AF1 GUSB SKAP2 IFIH1 FUS CLDN7 BCAP31 CAP1 NMT1 UGCG GUK1 NOLC1 GRB2 RPS6KA1 METAP2 PDCD5 UPF3B IGF2R NMB ATP5G2 RPA2 KCNK1 TGIF2-C20orf24///C20orf24 GAPDH HIST2H4B///HIST4H4///HIST2H4A///HIST1H4L///HIST1H4E///HIST1H4B///HIST1H4H///HIST1H4C///HIST1H4J///HIST1H4K///HIST1H4F///HIST1H4D///HIST1H4A///HIST1H4I SNRPA CCT6A SNX10 CRK MRPL23 ETFA EGFL6 WSB2 ITGB2 MRPS15 TFRC ERBIN DGKA IRX5 HDGF KARS PTPN12 UBE2L6 ACTR3 LAGE3 AHSA1 MIR7703///PSME2 GPX4 LCP1 NDUFV2 UBAC1 PLEK MED6 PPAT IGSF3 AGFG1 LCK STK17A PMF1 MYB SRSF1 PLK4 CDK5 PRSS8 SLC1A5 AKR1A1 PNO1 HSP90AB1 CYB5R4 PCBP1 JUP ABCC5 BAG6 SRSF4 MIR8085///BCL3 TMEM135///DNAJB6 ALG3 TNFRSF10B TNFRSF21 GSK3B NAP1L4 TNFAIP2 TRIM28 PSMC3IP UNG FANCA IDH3G POLD1 LAD1 BAZ1A IARS MAPK9 ATP6V1G2-DDX39B///SNORD84///DDX39B SUPT16H HLA-F PSMB7 CHEK1 RNF5P1///RNF5 MIR4640///DDR1 POLR3K SLX1B-SULT1A4///SLX1A-SULT1A3///SULT1A4///SULT1A3 SQRDL NARF GRN PTPN6 NUP50 CDK2AP2 MNDA PLA2G7 PARP2 CLDN1 MRPL3 CUL1 TKT ILF3 THEMIS2 PAQR4 KLF5 NDUFB4 TMEM2 ACOT9 HCK ADRM1 HIST1H2BH CYCS AKAP1 UBE2Q1 LOC101929240///SNRNP200 ODC1 SAP18 FMR1 PTPRK POLD2 CNOT8 MIR6758///MARS RFXANK ABCF1 RNPEP BCAT2 MIR6805///RPL28 MRPL12 ZNF165 MIR4745///PTBP1 RNASEH1 YY1 CREB1 NUDC POLE PLK1 MVP BUB3 SF3B4 TUBB AATF SMPDL3B PRKCD CASP8 H2AFY CENPI LDLR HSPB11 TMC6 LOC101927137///CCT3 HADHA TNFAIP1 DIAPH1 ADAR MIR1292///SNORD110///SNORD86///SNORD57///NOP56 TPR NUDT21 PAK6 NIF3L1 CKAP4 TNFAIP8 PTER CSNK2B UBE2D3 SLC35B1 CSNK1A1 SLC9A3R1 PSMB10 POPDC2///COX17 FXYD5 IL2RG CAD PGK1 EIF6 AK6///TAF9 VRK2 ACO2 LSM7 KDELR1 VDR SMARCC1 RHOA RFC2 SCAF11 PPP1R7 SELL PPT1 APITD1-CORT///APITD1 DUT MMD HLA-C MYH9 SPAG9 SFPQ SMC2 MIR1908///FADS1 PTMA CCNL1 PTBP3 RAD1 PPP2CA NUP153 HNRNPR FAF1 ARFGEF1 IL4R ENOSF1 PNKP XPO1 PRPS1 COX6A1 GNG5 NFKB1 UBA1 GLA PSMC4 PSMC2 ATP2C1 SPHK1 TFG F8A3///F8A2///F8A1 DDX21 FDPS RAB22A GCHFR TOR3A HSP90AA1 EIF3I DNM1L EZR SNX4 PRPF8 RNF114 LOC642846///DDX12P///DDX11 ORC6 HSPD1 POLQ TLR2 NFE2L2 HIRA CPNE1 NMD3 RAD23B CBX3 TUBB4B SHMT2 NPL ELAVL1 LY6E MIR3620///ARF1 BID DPP3 CBFB ARPC3 TYK2 ADAM8 AKT1 ARRB2 ARID1A SRM SSBP1 IQGAP1 NDUFB5 FBXW2 PLXNA2 CFL1 SRSF10 SUMO1 ESPL1 NEU1 COPE MSL3 VCP PPP4C LHX2 ALDH18A1 MAT2B JTB CDK16 SLC25A3 RNF13 ECH1 CBS ATF5 RCC1 GPS1 REC8 PHB2 MED1 NUCB1 CHD1 GTF3C3 COPB2 MAPK13 NFE2L3 MTCH2 KEAP1 PSMD5 LYN ACTB CCT7 ANP32A PPID YWHAB APEX1 OAZ1 RBBP8 LMNA TIGAR RPL8 PITPNC1 AGPS WBSCR22 UBE2M GRSF1 ACP1 SLC5A3 ETV7 CARHSP1 FXR1 CASP6 RAD51C MDH2 CHFR COTL1 ORC1 RPA1 PRPSAP1 PITPNM1 EIF3CL///EIF3C RECQL4 MDH1 TAP2 LASP1 NCBP2 XRCC1 SSRP1 XAF1 RPN1 PHTF1 RPS19 CRLF3 HDAC2 NDUFS2 CAPRIN1 DHX9 PTGES3 TIMM23B///TIMM23 ALAS1 SUMO3 CBX5 PEF1 GOLPH3 ITGA2 FANCG CASP3 CBX1 EML4 CDH1 LOC101927705///P4HA2 MIR664B///SNORA56///DKC1 SRP9 ATP6V1G1 TRIM38 PQBP1 USP39 VPS72 SPTAN1 DAP RSRC2 ARHGEF16 PPP6C MSRB1 CPSF6 LBR GLO1 LTB B3GNT3 FAM60A LIMA1 KIF20B COX8A MED15 SNRNP40 TMSB10 CEBPA SLC15A3 DDX23 DAP3 UBE2G2 AP3B1 ACTN4 NDUFS6 OGDH SNRPC MPV17 TOMM40 DDX41 PIK3CB NASP M6PR///PHC1 SNRPF CORO2A ZNF195 IDH3B CLK2 AK2 SS18 DYRK2 TAF12 MAP3K7 MRE11A TRA2B BCAS2 GNPDA1 SREBF1 SP140 AARS TCEB1 COX5A SNRPD2 HNRNPM BRD7 COMT TRAPPC3 NNT TUFM GRB7 RUVBL2 LOC101927180///ATP6V1F TSEN34 UBL4A EXOSC2 MRPL17 HPS5 CUX1 HIST1H2BC///HIST1H2BI///HIST1H2BE///HIST1H2BF///HIST1H2BG PCNT ATP5G1 CERS2 CHEK2 MAPKAPK5 ACACA NDUFA7 CLN3 POLA2 IPO7 KIF2A UBA2 TUBGCP4 HNRNPD MTX2 CHKB-CPT1B///CPT1B EIF3J HSPA9 SLC7A7 GJA9-MYCBP///MYCBP PAICS PTP4A2 YIF1A AIP RAB27A PSMD7 SDHAP2///SDHAP1 PPP2R1A RHOG MNX1 EIF2B1 HNRNPA3///HNRNPA3P1 CNOT7 PRDX4 DAZAP1 NUDT21 LDHC TAF15 CYTH2 KPNB1 PRPF3 FGFR1OP VASP ARMC2-AS1///ATP5J2 PSMF1 SNN NONO CDC123 CTNNBL1 MYL12B///MYL12A ARPC5L TUBB3 C11orf58 OAZ2 RAC2 FUBP1 HSD17B10 VAPB GALK2 MAN2B1 HP1BP3 EXOSC10 PDHA1 TRAF3 SCAMP3 DDX11 UFD1L LOC101929876///LOC100996747///RPS26P11///RPS26 ERP29 GCA LSM3 GTF2IRD1 MIR6778///SHMT1 MRPS30 DYNLT1 NRDC LOC101928623///HARS GBAP1///GBA MLF2 WBP11 UQCRHL///UQCRH FIBP IL1RAP TCOF1 HSBP1 CPOX DHPS HEXIM1 PSEN1 COX6B1 COPS5 SNRPG HOXD10 NAA10 RBM8A MIR6824///SLC26A6 CNIH1 HIRIP3 BATF DPM1 HPCAL1 UTP6 APMAP PAPD7 STOML2 KTN1 RCN2 ATP5G3 PUDP SLC3A2 CLTC RBM39 CHCHD2 PDSS1 CDC5L PSME1 LSM6 DDB2 SNX5 OXCT1 B2M GRP RELB ENSA U2AF2 GNL2 MRPL58 BAD CNOT3 IRF3 NDUFS1 HINT1 MAPK1 SNF8 STX4 FAM50A PRPF18 POLR2D DAG1 SOAT1 HUWE1 CLPP SNU13///ANXA2 PSMB5 ACP2 BRD3 ATP5B NOL7 POP7 GTF2A2 MIR1236///NELFE NXT1 SAP30BP ICMT AKAP8L PIGT FANCE DDX56 MIR1248///SNORA81///SNORA4///SNORD2///SNORA63///EIF4A2 CNTRL VRK3 BCL7B PSMC1 TOP1 TARBP1 NDUFAB1 MAN1B1 UPF1 TFAM RNF4 RBL1 FOXK2 YARS TRIM26 ZNF45 XRCC5 PDK1 STK38 MCCC2 ENOPH1 DNM2 PSMB4 SNRPD3 CCT4 AIMP1 CSNK1G3 ARPP19 TACO1 ERH KLHL12 TXNL4A CASP10 WRAP73 SHB OXA1L SNRPA1 PUS1 PPP1CC RPL26L1 NDUFA8 LPCAT3 KPNA1 RAC1 EXOSC5 ZNF706 EMC8 MAP3K13 SLC25A4 PTK2 NCF4 SSB HAUS7///TREX2 FLOT2 STRN4 NUPL2 IGSF6 PAK1 RPP38 LPAR2 PDP1 CCHCR1 RBM14-RBM4///RBM4 GTF2H4 ARIH2 POLD4 SMARCB1 PTTG3P RNPS1 LSM8 ATF6 POP4 TOR1A MFAP1 RARS SETDB1 METTL13 PIGA COPS3 YKT6 BRCC3 MIR3652///HSP90B1 RAB32 DFFA SENP2 TMPO PRRC2A EWSR1 PPP1R11 DIEXF NFKBIB CCNF GABPB1 SUV39H1 RGS19 UQCR10 LOC101927733///HMGN1 CCHCR1 MAPKAPK2 MSH3 GMIP HCCS SRRT PPM1A MKNK1 NCKAP1L THEM6 TIMM17B TNPO3 ABT1 DIABLO HCFC1 DEDD KHDRBS1 MAP2K1 MAPKAPK5-AS1 PITPNB COASY DGUOK AUP1 SH2D3A RBM14 TBC1D3P1-DHX40P1///RNFT1 PDE6D DCAF7 DUS4L E2F3 CIDEB LARP1 PRR13 CSTF2 POLR1C ORC3 TBCC TBK1 RELA APEX2 ZNF200 PES1 SLC20A2 MED7 SIPA1 ARAF CSNK1G1 TIMM44 LOC101060373///NOMO3///NOMO2///NOMO1 TSSC1 SLC2A6 AIMP2 CIAO1 TPRA1 WDR1 HNRNPC NAIP CTCF PMS2P3 ZNF354A DNAJA3 NT5C METTL3 TNFRSF25 ADAM15 SOX30 ERF POLG E2F1 PAXBP1 EIF4H HAPLN1 OSGEPL1 AAMP UCN IFNGR2 CPSF4 YEATS4 XRCC4 PRIM2 ZFY///ZFX |
