# Supplementary material for: Identification and validation of novel biomarker TRIM8 related to cervical cancer
Source: Front Oncol. 2022 Oct 24;12:1002040. doi: 10.3389/fonc.2022.1002040 (PMC9638460; doi:10.3389/fonc.2022.1002040)
Supplement: Supplementary file 2 [file Table_2.docx]

**Supplementary Table 2 Ubiquitination and Autophagy related genes**

|  | Gene name |
| --- | --- |
| Ubiquitination-related genes | HDAC6 UBE2DNL PDCD6 WASHC1 RNF212B UBA2 SAE1 HUWE1 TRIM10 FEM1B TRIM28 RNF41 PRMT3 CDK5 TRIM13 TOPORS DCAF7 CDK9 SPRY2 STUB1 UBE4B TRAIP MAEA MARCHF6 KLHL41 PCGF3 TRIM22 HMG20B HMG20A ANAPC10 RACK1 PIAS3 UBAC1 ARIH2 MAD2L2 TRIM38 UBE2E3 LRRC41 APPBP2 ATG7 UBD TRIM3 RBCK1 IVNS1ABP RFPL3 RFPL2 TRAF3IP2 MALT1 OS9 RAB40B MID2 WWP1 WWP2 UBE2C TRIM31 GLMN FAM107A RNF139 RNF24 BIRC8 CHP1 KLHL2 MED8 PARK7 GABARAP U2AF2 RNF13 DTX2 TRIM9 RNF157 FBXO32 MARCHF3 FBXO17 CISH UHRF2 RNF166 ERCC8 KLHDC3 RFFL TRIM6 UBE2J2 C10orf90 ANAPC16 TRIM64 LRRK2 ANKRD9 LRR1 KLHDC1 SOCS4 LEO1 DCUN1D3 FBXO27 ASB17 RNF19B TRIM43 UBR3 KLHL40 TRIM71 UBE2QL1 TRIM40 TRIM50 ASB10 RNF183 AMER1 ASB11 ASB5 ASB6 ASB7 ASB8 ASB9 TRIM69 CRY1 UBE2F NEURL2 MIB2 RAB40A ASB15 ASB14 ASB12 HECTD2 TTC36 PRICKLE1 FBXL14 RNF151 KCTD11 C18orf25 TICAM1 ZNRF4 ZNF738 UBE2U RC3H1 DCST1 RNF187 CTNNB1 COPS9 COMMD1 ZSWIM2 WDSUB1 DTX3L RNF38 SH3RF2 RNF145 RNF217 CBLL2 DAXX MARCHF10 SDE2 DDB1 DDB2 DAW1 UBXN2A DDX3X RNF168 TRIM60 CDC20B RNF133 UBE2L5 DTX1 E4F1 PHC1 EGR1 EGR2 DTX3 UBR1 NSMCE1 FAAP20 RNF215 KCTD6 FBXO45 CENPX EPAS1 TRIML2 AKT1 EYA1 FANCA FANCC FANCE FANCB FANCF FANCG RNF152 MARCHF8 RNF182 LNX2 ZNRF2 FKBP1A UBOX5 FOXF2 TRIM32 PAXIP1 KLHDC10 FBXO21 PDZRN3 KDM1A WDTC1 ZSWIM8 CAND2 HECW1 MYCBP2 TRIM35 CUL9 DCUN1D4 FBXL7 FBXO28 DTX4 MINAR1 KLHL18 FBXW11 MGRN1 UBR2 TRIM2 USP22 NEDD4L SASH1 JADE2 UBR4 UFL1 SIRT1 RYBP SUZ12 PRAME RASD2 GTPBP4 KLHDC2 MKRN1 MKRN2 RUSC1 CBLC HSPBP1 PPIL2 CDC26 PELI3 ABL1 FYN KCTD13 BXO33 RNF169 RNF144B RNF214 SHPRH FBXO7 ARIH1 FBXL2 HECTD1 ANAPC13 DCAF12 DCAF13 TRIM58 RNF19A RCHY1 WWTR1 RWDD3 RNF167 ZNF451 LTN1 SENP6 HERC4 DCAF4 WSB1 TRPC4AP IRF2BP1 FBXW2 PTPN22 FBXL21P FBXL3 FBXO2 FBXL6 FBXL5 KLHL3 FBXW8 FBXO25 FBXO24 TSPAN17 FBXO22 FBXO10 FBXO9 FBXO6 FBXO5 FBXO4 FBXO3 GNL3 MAGEA2B AMFR RNF11 MTBP RNF115 KLHL20 GCLC MOCS3 UBE2S PRPF19 RABGEF1 RAB40AL TRIM49B KCTD21 HECTD4 SIAH3 HERC2P3 FBXL22 NHLRC4 ANGPT1 RNF227 RNF149 RNF212 RNF175 RNF180 NSMCE2 FBXO43 TRIM59 GPS2 UBE2T MYLIP UHRF1 GSK3A ANAPC2 ANAPC4 UBQLN1 SOCS7 HIF1A UBE2K KLHL10 BIRC2 BIRC3 DNAJB2 DNAJA1 HSPA1A HSPA1B HSPA5 XIAP HSP90AA1 HSP90AB1 KLHL17 SPOPL TRIML1 NCCRP1 IFI27 RFPL4A SH3RF3 CTU2 TRIM23 SVBP TRIM73 TRIM74 CENPS NHLRC1 RNF148 SUMO4 NHLRC3 UBE2NL TRIM77 KBTBD13 SUMO1P1 TRIM61 TRIM75P LIMK1 TRIM49D1 MED11 LMO7 ASB18 LNPEP RNF223 ARNT MIR101-1 MIR138-1 ARRB1 MAD2L1 ARRB2 SMAD7 MAGEA2 MDM2 MEFV PRAMEF6 MARCHF11 TRIM37 NEDD4 NEDD8 NEUROD2 NFE2L2 NFX1 CNOT4 TRIM72 RNF165 PRKN DCAF8 RNF141 MED31 UBXN1 NOSIP TRIM17 ASB3 RLIM RNFT1 VPS28 CRBN HERC5 ANKRD39 RNF181 TBC1D7 MARCHF2 BFAR MEX3C WAC FZR1 UBR5 ANAPC5 ANAPC7 MAGEC2 RNF138 UBE2J1 UFC1 DTL ANAPC11 ZC3HC1 SIRT7 HDAC7 UFM1 PIAS4 TRIM33 UBE2D4 ASB1 ASB4 NUB1 ASB2 FBXO40 PEX12 PIN1 BCL11A BTBD1 PLK1 PML TRIM34 SEPTIN4 DCUN1D1 FBXW5 ANKIB1 TOLLIP RNF216 NEURL1B RC3H2 RNF186 MAGEL2 GNL3L NDFIP2 PAF1 MARCHF5 TRIM44 RNF111 PPIA KCTD9 TRPM4 KLHL24 BSPRY WBP1L FBXL12 DCAF16 BCOR RNF43 UBE2R2 RNF125 PINX1 HERC6 MARCHF1 DET1 RNF31 WDR70 FANCL TRIM68 UBR7 RFWD3 MSL2 RNF220 DCUN1D2 FANCI TRIM62 UBA6 UBE2W FBXW7 RNF121 PEF1 TRIM36 FEM1A UBE2Q1 OTUB1 AMBRA1 G2E3 RNF126 CHFR INAVA PRKCE RNF130 PRKCG VPS11 DCAF6 CAND1 HDAC8 WSB2 RNF114 ZC4H2 KLHL9 KLHL7 MAPK9 NSMCE3 RNF20 PSEN1 TRIM39 RAD18 KCMF1 FEM1C ATXN7L3 TULP4 PCNP TRIM49 SMURF1 TRIM54 PSMD10 PELI2 PELI1 ZMIZ1 PTEN CBX8 BIRC6 RNF150 ADGRB1 HECW2 HACE1 MIB1 KLHL42 ARRDC3 KLHL8 MARCHF4 WDR48 VPS18 SH3RF1 UVSSA RNF213 KIAA1586 FANCM TRIB3 RAB40C CCNB1IP1 BARD1 RAG1 RAG2 RANBP2 RANGAP1 RBBP6 SENP2 KLHL12 BCL2 RELA TRIM27 RFPL1 RING1 RNF2 RNF4 RNF5 RNF6 RPL5 RPL11 RPL37 RPS2 RPS3 RPS7 RPS15 RPS20 RPS27A RNF123 UBE2O SEL1L MAGEF1 NOD2 IFIH1 BLMH IRF2BPL PJA1 TRIM64B TRIM49C RNF25 COP1 NXN GMCL2 AKTIP KLHL25 ATG3 ARRDC5 TRIM64C ANAPC1 RNF225 UNKL SMURF2 SIAH1 RMND5B SIAH2 RMND5A BMI1 FBXL17 MARCHF7 SKP1 PINK1 SKP2 UBE2Z TRIM43B HLTF DDRGK1 SUMO3 SUMO2 SOX4 BRCA1 TRIM21 STX1A TAF1 ELOC ELOB PPP1R11 BUB1B TGFBR1 TSPO THOP1 TNFAIP1 TNFAIP3 TPP2 TRAF1 TRAF2 TRAF3 TRAF5 TRAF6 TSG101 EIPR1 TTC3 RNF208 TRIM49D2 RFPL4AL1 UBA52 UBB UBC UBA1 UBA7 UBE2A UBE2B UBE2D1 UBE2D2 UBE2D3 UBE2E1 UBE2E2 UBE2G1 UBE2G2 UBE2H UBE2I UBE2L3 UBE2N UBE2V1 UBE2V2 UBE3A SUMO1 UCHL3 USP4 VCP VHL WFS1 PTTG1IP MKRN3 MKRN4P PCGF2 ZBTB16 TRIM25 TRIM26 RNF112 RNF113A LAPTM5 RNF103 USP7 OTUB2 DDA1 GID4 TRIM48 RNF26 DERL1 PHF23 FBXL15 DCAF10 BIRC7 CDC73 RNF128 MUL1 OBI1 AIMP2 HECTD3 ASB13 KLHL36 FBXO31 RNF122 CBLL1 UBA5 DCAF17 VCPIP1 SPSB1 RNF34 FBXO11 FAAP100 TRIM45 CDK5RAP3 KLHL15 SEL1L2 DCAF11 TNKS2 RNF39 CUL5 PRR7 NDFIP1 USP5 ZFP91 GAN FBXO38 TRIM11 TRIM8 URM1 TRIM7 RNF170 TRIM56 RNF146 SHARPIN CAMLG LZTR1 USP9X CAPN3 AXIN1 BRAP CDCA3 RASSF5 ATG10 ITCH KCTD10 SPRTN SPOP FSCB KBTBD7 FBXO30 USP44 PCGF6 ZNRF3 TRAF7 MED10 DCUN1D5 RNF135 PCGF5 SYVN1 NEURL4 CUL4B CUL4A CUL3 CUL2 CUL1 KBTBD8 TRIM55 TRIM63 KDM2B LNX1 SPSB2 OGT PCGF1 TRIM51 TRIM52 KLHL22 PARP10 FBH1 RNFT2 MASTL ZNRF1 ENC1 CBX4 TRIM5 TSPYL5 PIAS1 CDC14B CAV1 ABCB11 CBFB SOCS1 TNKS CBL CBLB CDC23 RIPK2 TRIM24 DCAF5 SOCS2 HDAC3 PER2 SPHK1 SQSTM1 CDC16 NAE1 TRIM4 BCL10 HERC3 HERC2 HERC1 BTRC TRIM15 KBTBD6 CCNF UBE3B RSPRY1 UBE3D SOCS3 RNF8 OTULIN KLHL13 CTU1 DCAF15 UBA3 MED30 UBE2M PIAS2 LRSAM1 ZNF598 SPSB3 TRIM41 TRIM47 NMI MTA1 RNF185 NEURL1 BUB3 ZBED1 ARRDC4 TMEM129 SPSB4 UBE2L6 ASB16 ARRDC1 UBE2Q2 MARCHF9 SOCS6 NEURL3 TRIP12 RPL23 UBE4A FBXO44 PLAA MED27 MED7 ATG5 NPEPPS BAG5 BAG2 RNF14 RNF7 ISG15 CTR9 SOCS5 DZIP3 N4BP1 UBE3C HERPUD1 DCAF1 HDAC4 RNF144A RNF40 KEAP1 CUL7 TRIM14 MED24 PJA2 AREL1 KLHL21 CDC20 RNF10 CDC27 MED12 CDC34 RBX1 CDC42 |
| Autophagy-related genes | BCL2L11 OPTN TRIM13 CALCOCO2 TRIM22 NOD1 TRIM38 KAT5 SPTLC1 NPRL2 CAMKK2 CERS1 PLK2 PIM2 MID2 WDR45 SNX18 PARK7 TRIM6 LRRK2 PLK3 SMCR8 SESN3 TICAM1 ADRB2 DAPK1 DCN FLCN TRIM65 ENDOG C9orf72 TRIML2 DHRSX RNF152 ATF6 RAB3GAP1 TRIM32 FOXO1 FOXO3 ATG2A LARP1 UFL1 SIRT1 SH3BP4 RAB3GAP2 FBXO7 SVIP GPSM1 HSPB8 SESN1 GNAI3 RUFY4 SLC25A4 TBK1 SLC25A5 GSK3A GSK3B HTT HIF1A HMGB1 HMOX1 TRIML1 STING1 IRGM IFNG IL4 KDR SNX30 MEFV PAFAH1B2 PRKN SH3GLB1 WAC SNX7 PIK3C2A PIK3CB PIP4K2A TPCN1 TRIM34 ATG16L1 WIPI1 RNF31 TRIM68 VPS13D PRKAA1 AMBRA1 PRKAA2 PRKD1 MAPK3 BAD ELAPOR1 TP53INP2 RALB TRIM27 SCOC ROCK1 MOAP1 NOD2 PINK1 BNIP3 BNIP3L TRIM21 STK11 SUPT5H MAP3K7 TSC1 TSC2 UVRAG VDAC1 PLEKHF1 TFEB FYCO1 EPM2A PIP4K2C ZC3H12A NPRL3 TRIM8 SESN2 PIP4K2B ULK1 IKBKG TRIM5 BECN1 SNX4 RIPK2 LACRT LRSAM1 MTDH ORMDL3 TP53INP1 SPTLC2 TMEM59 BAG3 DEPDC5 ULK2 TRIM14 |
